# Analysis of the preferences for splice codes across tissues

**DOI:** 10.1007/s13238-015-0226-5

**Published:** 2015-10-27

**Authors:** Tao Huang, Meng Wang, Yu-Dong Cai

**Affiliations:** College of Life Science, Shanghai University, Shanghai, 200444 China; Institute of Health Sciences, Shanghai Institutes for Biological Sciences, Chinese Academy of Sciences, Shanghai, 200031 China

**Dear Editor**,

Alternative splicing (AS) is a post-transcriptional process that can add complexity to proteome greatly by producing multiple different mature transcripts from the same pre-RNA (Black, 2003). Ever since its discovery, multiple kinds of different AS types have been identified, for example, exon skipping (or cassette exon), intron retention, alternative 3′/5′ splice site (SS) and so on (Wang et al., 2008). Among them, the exon skipping event is expected to be the most abundant (Black, 2003). By applying different kinds of large scale data, such as exon array and EST, lots of progresses have been made. Recently, with the development of high throughput RNA-sequencing technology, our knowledge about AS has been further exploded (Wang et al., 2008).

Four elements are primarily needed for splicing, which are the 5′ and 3′ SS (typical GT/GC at the 5′ end of intron, and AG at the 3′ end of intron), the branch site (usual a ‘A’ within intron near the 3′ SS), and the polypyrimidine tract which is an region enriched of pyrimidines (C or T) between branch site and 3′ SS. During the splicing process, several small nuclear RNAs and more than 100 proteins are incorporated, forming many associated complexes (snRNPs), the combination of which is also called “spliceosome” (Matlin et al., 2005). These snRNPs can recognize and bind with these sites, further mediate the removal of introns and the joint of exons.

In addition to these sites, plenty of other cis-acting elements or motifs have been identified to be associated with the splicing activity of genes. These elements or motifs, or splicing regulation elements (SREs), include well documented or predicted exon splicing enhancers and silencers (ESEs and ESSs), and intron splicing enhancers and silencers (ISEs and ISSs). Some clarified motifs such as YCAY clusters, CU-rich sequences and [U]GCAUG can bound directly with splicing factors, such as Nova, PTB, Fox proteins and so on, and promote or repress the splicing processes. At the mean time, hundreds of other motifs are predicted or experimentally supported to be associated with splicing with little or no relevant information of trans-acting factors.

Given such high abundance of splicing influencing elements, one may wonder how do they interact with each other, and function as a whole to determine the splicing or AS processes. Xiao, X. et al. (Xiao et al., 2007) found that several elements, such as 5′ SS with ESEs and others, evolve in a compensatory manner. Other works also indicated co-regulation among different cis- elements, together with trans- splicing factors (Wang et al., 2012). Another key question is that since AS is mostly tissue-specific, then how are these elements chosen, or used preferentially across different tissues? Castle, J. C et al. (Castle et al., 2008) used data of 48 human cell lines or tissues, and predicted a total of 143 “words” or short sequences that may contribute to tissue specific regulation of cassette exons. Barash, Y. et al. (Barash et al., 2010) further collected a comprehensive list of 1014 cis- features to meet this purpose.

However, this question still holds since former studies have limited power. Thus in this study, we analyzed the alternative splicing dataset from Barash, Y. et al. (Barash et al., 2010) with mutual information based feature analysis to further address it. We observed that for different tissues, the preferred features vary to some extent. Further analyses indicated that transcript structure features tend to be preferred universally. And finally, some of the features we identified were supported by existing reports, while others provided guidelines for further experimental studies.

For each tissue, we compiled six balanced non-alternative spliced (NAS) and alternative spliced (AS) datasets and on each dataset, the key features for discriminating NAS and AS cassette exons were identified with mRMR + IFS approach as described in Supplementary Materials. As shown in Fig. 1, the IFS curves were used to identify the key features on each dataset that achieved the highest MCC. The number of key features and their MCCs were shown in Table S3. In CNS, muscle, embryo and digestive tissues, the numbers of key features were 122, 391, 343 and 464, respectively. These features were shown in Table S4. We grouped the key features from each tissue into known motifs, new motifs, short motifs and transcript structure features as in Barash, Y. et al (Barash et al., 2010). The compositions of different feature groups in each tissue were shown in Fig. S3. And their average MCCs were 0.373 ± 0.019, 0.328 ± 0.034, 0.246 ± 0.016 and 0.268 ± 0.038, respectively. The preference of the key features may illustrate the splicing difference between tissues.

**Figure 1:**
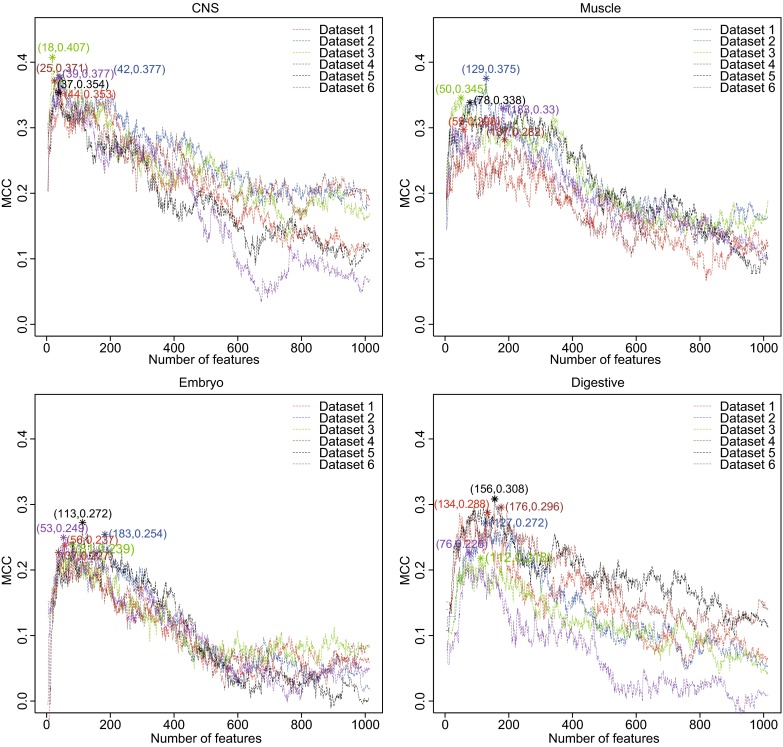
**The IFS curves in four tissues**. The x axis was the number of features and the y axis was the 10-fold cross validation MCC. The features achieved the highest MCC were selected as key features

Since AS often show tissue-specific manner, and that the abundances of splicing factors are not likely expressed equally across different tissues (Grosso et al., 2008), one may wonder how cis- splicing elements co-operate with these splicing factors within tissues. Note that several splicing elements, such as the 5′ splice site, 3′ splice site, branch site and polypyrimidine tract, are needed in almost all kinds of splicing processes (Black, 2003), it is thus expected that there must be other motifs contribute to tissue-specific manner, and the preference of some motifs should be different in different tissues.

Our results indicate that 45.8% of all identified features are found in one particular tissue, but not the other three ones (Fig. S4). We found that the binding of same splicing factor onto different regions is often associated with regulated splicing in different tissues. For example, the binding of Nova to upstream exon is often associated with AS in embryo; whereas its binding to the alternative exon exactly may be preferred in muscle.

We also found 35.9%, 14.6% and 3.7% of total features in 2, 3 and 4 tissue types respectively. This result reflects the complex situation of both similarity and inconformity among tissues. For example, the position of alternative GT is associated in both muscle and digestive tissues but not the other two; the length of alternative exon is associated in embryo and digestive tissue but not the other two. Our observation may provide valuable information for exploring such relationship of multiple tissue types.

The above result suggests that most features may be preferred in particular tissues, and that the occurrences of features vary. The occurrence of feature may reflect the weight for AS, features with high occurrences likely are more important than others. We thus ask whether the occurrences of all features types are equally or not.

We classified the features into 4 groups as well. As a result, we observed that for features with different occurrences, the compositions are significantly different (Fig. 2, chi-square test: *P* = 1.69 × 10^−5^). Specifically, the proportion of short motifs was lower for those identified within all 4 tissue types (comparing occurrence = 4 with the combination of other 3 groups: 10.7% vs. 44.2%); meanwhile, higher proportion of transcript structure features was observed for those with greater occurrence (comparing occurrence = 4 with the combination of other 3 groups: 28.6% vs. 5.3%). This result indicates that even though all groups of motifs contribute to alternative splicing, their usage among different tissue types varies: short motifs are likely used for regulated AS within partial tissue types; and features from transcript structures, though fewer compared with other groups, tend to be used universally.

**Figure 2:**
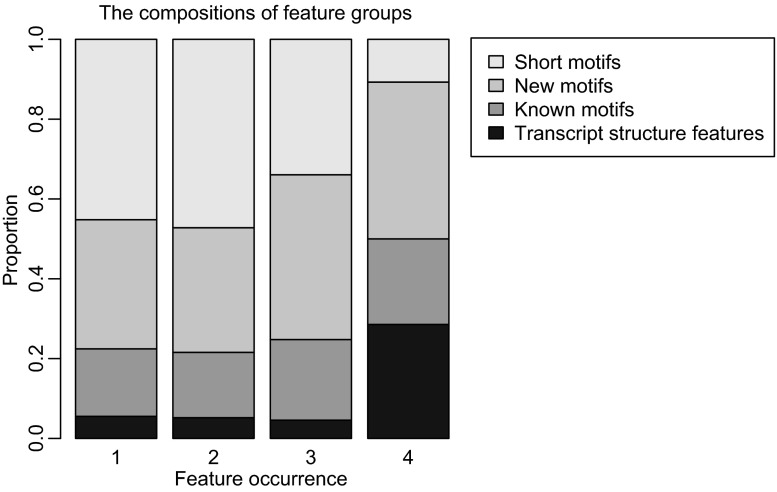
**The compositions of different feature groups with different occurrences**. The horizontal axis indicates the occurrences for features across tissues. “1” means the number of features that are only occurred in one tissue. “2” means the number of features that are discovered in two tissues and so on

Given the set of features that have been shown to be responsible for AS in these 4 tissue types, we next wonder whether our results are consistent with other studies. As a result, we found that many features we classified can be supported by former studies. For example, the position of alternative AG was found to be associated with AS (Gooding et al., 2006), and in our results, this feature is relevant with tissue-specific splicing in all 4 tissues. The downstream intron binding sites for Mbnl gene was found in our results and has also been reported (Faustino and Cooper, 2005). We also identified the binding sites for PTB, also intronic splicing silencers (Ashiya and Grabowski, 1997; Chan and Black, 1997).

On the other hand, we further identified several features that previously have no or limited evidence associated with AS. For example, short sequences such as CCC and TTG within alternative exon, CAG within upstream constitutive exon. New motifs such as TGTCT, TGCCTTT within downstream intron regions were also found though having been predicted with few supports to be associated with splicing (Yeo et al., 2007). Thus, our study actually provide useful hints for further research of AS.

## Electronic supplementary material

Supplementary material 1 (PDF 1168 kb)
