# Supplementary material for: Analysis of the preferences for splice codes across tissues
Source: Protein Cell. 2015 Oct 27;6(12):904–7. doi: 10.1007/s13238-015-0226-5 (PMC4656204; doi:10.1007/s13238-015-0226-5)
Supplement: Supplementary file 1 — Supplementary material 1 (PDF 1168 kb) [file 13238_2015_226_MOESM1_ESM.pdf]

Supplementary Text for "Analysis of the preferences for splice codes across tissues"

Tao Huang[2*#], Meng Wang[2#], Yu-Dong Cai[1*]

[1] College of Life Science, Shanghai University, Shanghai 200444, P.R. China

[2] Institute of Health Sciences, Shanghai Institutes for Biological Sciences, Chinese Academy of Sciences, Shanghai 200031, P.R. China

Methods

Dataset

We download the alternative splicing data in central nervous system (CNS), muscle, embryo, digestive tissues from http://genes.toronto.edu/wasp/index.php?mode=dataset. This dataset was compiled by Barash et al. (Barash et al., 2010) based on the alternative splicing microarray data from Fagnani et al. (Fagnani et al., 2007) which used probes to detect whether the known alternative splicing events of cassette exon happen in a specific tissue. Barash et al. analyzed the inclusion value matrix of 3,665 cassette exons in 27 tissues which were grouped in four tissue types, i.e. CNS, muscle, embryo, digestive tissues, with a model-based Expectation-Maximization (EM) algorithm to determine the increased exon inclusion, increased exon exclusion and no change probabilities (Barash et al., 2010). The status with greatest probability was assigned as the status of this cassette exon in this tissue type. This dataset has been widely used in many studies (Leung et al., 2014; Xiong et al., 2011) and became a well-recognized benchmark dataset for alternative splice researches.

To simplify the problem, we only try to predict whether a cassette exon will be alternative spliced or not. The numbers of these two kinds of cassette exons were shown in **Table S1**. The numbers of shared alternative spliced exons among four tissues were shown in **Figure S1**. To discriminate these two types of cassette exon, 1,014 features from Barash et al. (Barash et al., 2010) were adopted to represent the cassette exon. The features can be categorized into four types: 171 known motifs, 326 new motifs, 460 short motifs and 57 transcript structure features. The known motifs were reported to be near tissue regulated exons or associated with splicing factor binding preferences in literature (Barash et al., 2010). The new motifs were motifs with weak or no known evidence for roles in tissue-dependent splicing (Barash et al., 2010). The short motifs were region-specific 1-3-nucleotide (Barash et al., 2010). Transcript structure features characterized the spliced transcripts, such as exon and intron lengths (Barash et al., 2010). The complete feature list can be found in **Table S2**.

Identify the features for tissue specific splicing of cassette exon

As shown in **Table S1**, most cassette exons were not alternative spliced. The number of non-alternative spliced cassette exons is roughly six times of the number of alternative spliced cassette exons. Therefore, we applied the procedures shown in **Figure S2** to reconstruct datasets with balanced non-alternative spliced cassette exons

and alternative spliced cassette exons, and analyzed the key features for discriminating these two types of exons on each dataset.

First, we equally divided the non-alternative spliced cassette exons into six parts and each part has similar number of non-alternative spliced cassette exons as the alternative spliced cassette exons. By combining the same alternative spliced cassette exons with different non-alternative spliced cassette exon parts, we compiled six datasets. In each dataset, the alternative spliced cassette exons were the same, i.e., all alternative spliced cassette exons in that tissue. But the non-alternative spliced cassette exons were different and had similar sample size with the alternative spliced cassette exons.

Then, on each balanced dataset, we utilized mRMR+IFS approach to identify the important features for predicting alternative spliced cassette exons. The mRMR+IFS approach will be described in the following sections.

At last, the identified features on each datasets were combined.

mRMR (minimal Redundancy Maximal Relevance) feature ranking

To rank the important features that can help the classification of alternative spliced cassette exons and non- alternative spliced cassette exons, we applied mRMR (minimal Redundancy Maximal Relevance) (Peng et al., 2005) which is a widely used in bioinformatics (Niu et al., 2013; Zhao et al., 2013; Zhou et al., 2015). It considered both the redundancy between features and the relevance between features and exon classes.

Let's use Ω, Ω_s and Ω_t to represent all N features, the selected m features, the to-be-selected n features. The relevance of the feature f from Ω_t with exon class c is measured with mutual information (Huang and Cai, 2013; Sun et al., 2012) (I):

$$D = I(f, c) \quad (1)$$

And, the redundancy between feature f and the selected features is

$$R = \frac{1}{m} \sum_{f_i \in \Omega_s} I(f, f_i) \quad (2)$$

The mRMR function is to obtain the feature f_j from Ω_t with maximum relevance with exon class c and minimum redundancy with the already-selected features and it is as following:

$$\max_{f_j \in \Omega_t} \left[I(f_j, c) - \frac{1}{m} \sum_{f_i \in \Omega_s} I(f_j, f_i) \right] (j = 1, 2, ..., n) \quad (3)$$

With N round of mRMR evaluation, all features can be ranked:

$$S=\left\{f_1^{'}, f_2^{'},..., f_h^{'},..., f_N^{'}\right\} \quad (4)$$

The top feature with a smaller index h is more important for discriminating different exons.

IFS (Incremental Feature Selection)

The ranked feature list needs to be further analyzed to determine the cutoff of how many top features should be selected. To do so, we applied IFS (Incremental Feature Selection) (Li et al., 2014; Shu et al., 2014; Yang et al., 2014; Zhang et al., 2015a). During IFS, we tested all candidate feature set $S_i=\{f_1, f_2,..., f_i\}\,(1\le i\le N)$ which included the top i features. And the feature set with highest 10-fold cross validation (Chen et al., 2014; Cui et al., 2013; Yang et al., 2014) Matthews's correlation coefficient (MCC) (Chen et al., 2015; Zhang et al., 2015a) was selected.

To construct a classifier to evaluate the classification performance of a feature set, we used a support vector machine (SVM) based method, SMO (sequential minimal optimization) (Zhang et al., 2015b) in Weka (Frank et al., 2004). The default parameters of SMO in Weka were adopted. The complexity constant was set to be 1, the tolerance parameter was set to be 1.0e-3, the epsilon for round-off error was set to be 1.0e-12, the exponent to use was set to be 1.0 and the kernel to use was poly kernel.

References

Barash, Y., Calarco, J.A., Gao, W., Pan, Q., Wang, X., Shai, O., Blencowe, B.J., and Frey, B.J. (2010). Deciphering the splicing code. Nature 465, 53-59.

Chen, L., Chu, C., Huang, T., Kong, X., and Cai, Y.D. (2015). Prediction and analysis of cell-penetrating peptides using pseudo-amino acid composition and random forest models. Amino Acids.

Chen, L., Lu, J., Zhang, N., Huang, T., and Cai, Y.D. (2014). A hybrid method for prediction and repositioning of drug Anatomical Therapeutic Chemical classes. Mol Biosyst 10, 868-877.

Cui, W., Chen, L., Huang, T., Gao, Q., Jiang, M., Zhang, N., Zheng, L., Feng, K., Cai, Y., and Wang, H. (2013). Computationally identifying virulence factors based on KEGG pathways. Mol Biosyst 9, 1447-1452.

Fagnani, M., Barash, Y., Ip, J.Y., Misquitta, C., Pan, Q., Saltzman, A.L., Shai, O., Lee, L., Rozenhek, A., Mohammad, N., *et al.* (2007). Functional coordination of alternative splicing in the mammalian central nervous system. Genome Biol 8, R108.

Frank, E., Hall, M., Trigg, L., Holmes, G., and Witten, I.H. (2004). Data mining in bioinformatics using Weka. Bioinformatics 20, 2479-2481.

Huang, T., and Cai, Y.-D. (2013). An Information-Theoretic Machine Learning Approach to Expression QTL Analysis. PLoS ONE 8, e67899.

Leung, M.K., Xiong, H.Y., Lee, L.J., and Frey, B.J. (2014). Deep learning of the tissue-regulated splicing code. Bioinformatics 30, i121-129.

Li, B.Q., You, J., Huang, T., and Cai, Y.D. (2014). Classification of non-small cell lung cancer based on copy number alterations. PLoS ONE 9, e88300.

Niu, B., Huang, G., Zheng, L., Wang, X., Chen, F., Zhang, Y., and Huang, T. (2013). Prediction of substrate-enzyme-product interaction based on molecular descriptors and physicochemical properties. Biomed Res Int 2013, 674215.

Peng, H., Long, F., and Ding, C. (2005). Feature selection based on mutual information: criteria of max-dependency, max-relevance, and min-redundancy. IEEE Trans Pattern Anal Mach Intell 27, 1226-1238.
Shu, Y., Zhang, N., Kong, X., Huang, T., and Cai, Y.D. (2014). Predicting A-to-I RNA Editing by Feature Selection and Random Forest. PLoS ONE 9, e110607.
Sun, L., Yu, Y., Huang, T., An, P., Yu, D., Yu, Z., Li, H., Sheng, H., Cai, L., Xue, J., *et al.* (2012). Associations between ionomic profile and metabolic abnormalities in human population. PLoS ONE 7, e38845.
Xiong, H.Y., Barash, Y., and Frey, B.J. (2011). Bayesian prediction of tissue-regulated splicing using RNA sequence and cellular context. Bioinformatics 27, 2554-2562.
Yang, J., Chen, L., Kong, X., Huang, T., and Cai, Y.D. (2014). Analysis of Tumor Suppressor Genes Based on Gene Ontology and the KEGG Pathway. PLoS ONE 9, e107202.
Zhang, N., Huang, T., and Cai, Y.D. (2015a). Discriminating between deleterious and neutral non-frameshifting indels based on protein interaction networks and hybrid properties. Mol Genet Genomics 290, 343-352.
Zhang, P.W., Chen, L., Huang, T., Zhang, N., Kong, X.Y., and Cai, Y.D. (2015b). Classifying ten types of major cancers based on reverse phase protein array profiles. PLoS One 10, e0123147.
Zhao, T.H., Jiang, M., Huang, T., Li, B.Q., Zhang, N., Li, H.P., and Cai, Y.D. (2013). A novel method of predicting protein disordered regions based on sequence features. Biomed Res Int 2013, 414327.
Zhou, Y., Zhang, N., Li, B.Q., Huang, T., Cai, Y.D., and Kong, X.Y. (2015). A method to distinguish between lysine acetylation and lysine ubiquitination with feature selection and analysis. J Biomol Struct Dyn, 1-12.

Supplementary Figures

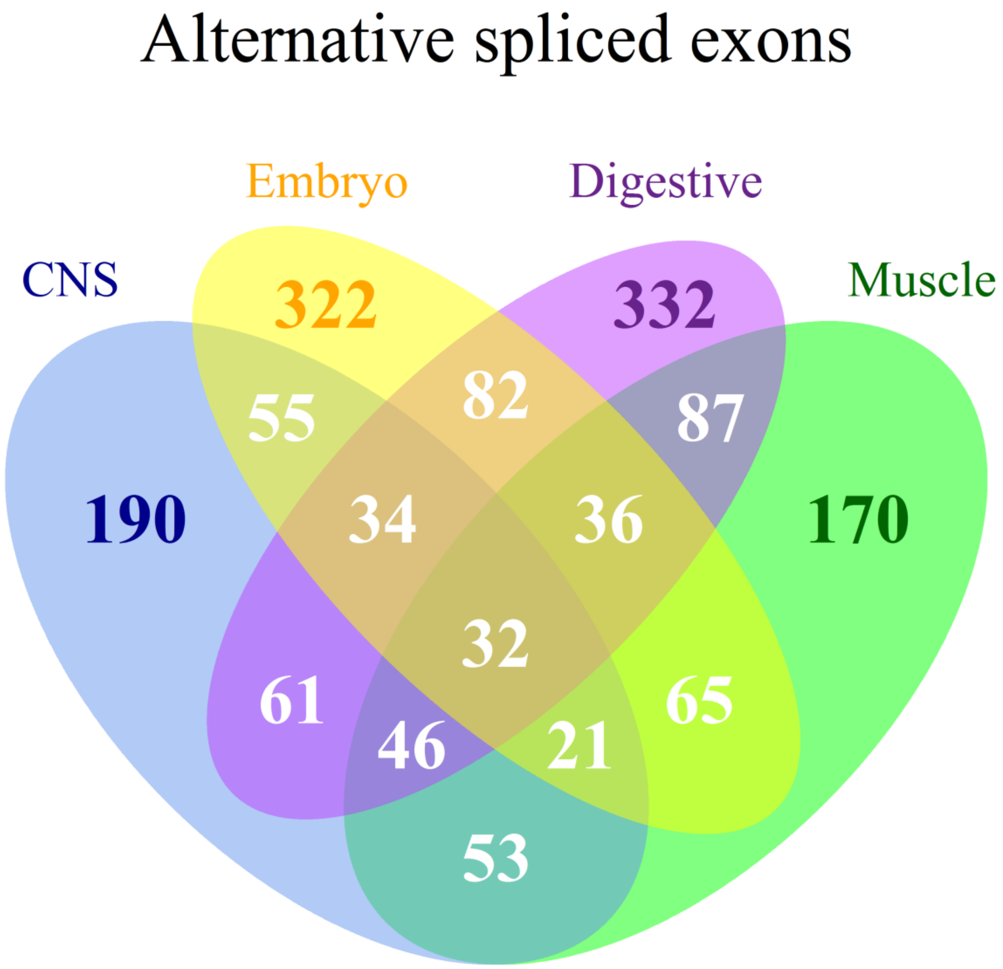

Figure S1 - Venn diagram for shared AS exons among four tissues

The numbers of tissue specific AS exons and shared AS exons were shown.

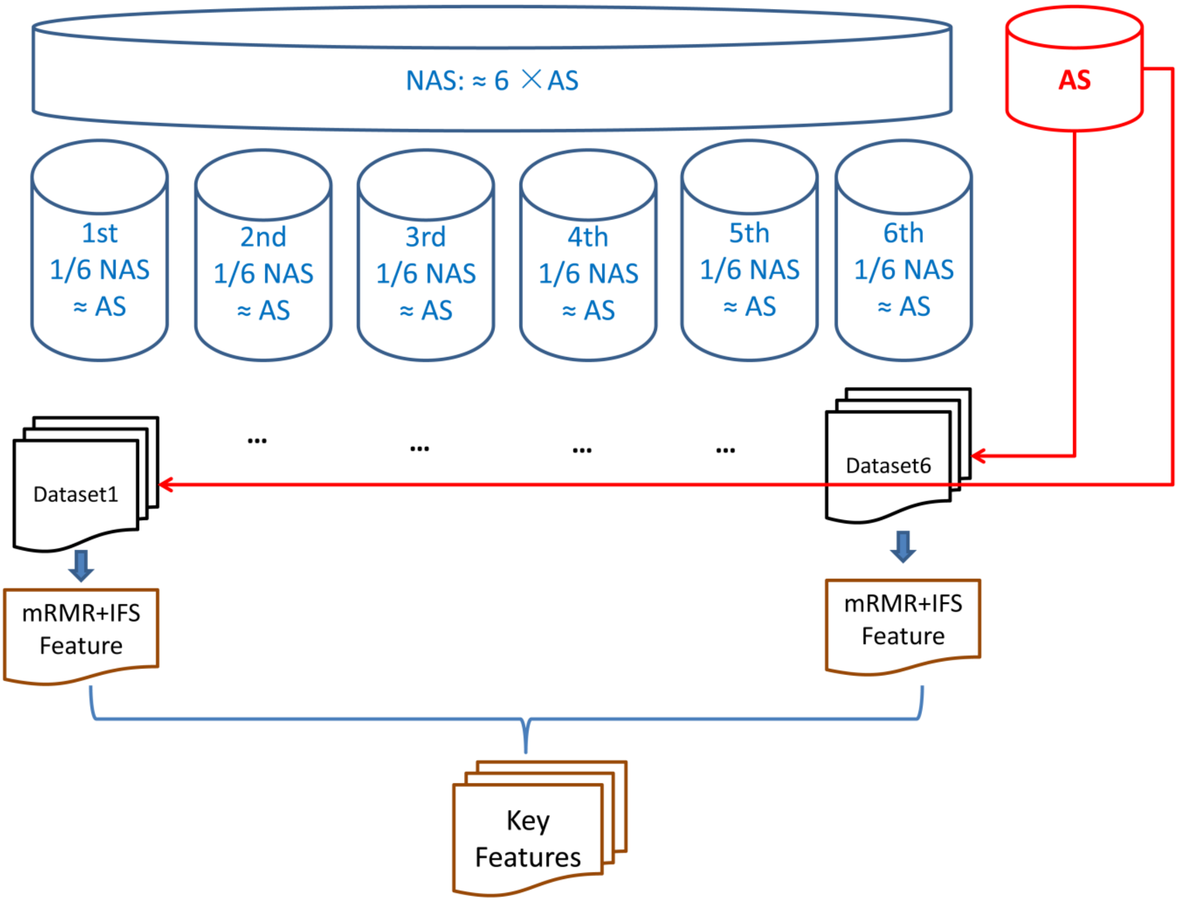

Figure S2 - The workflow of identifying key features for discriminating non-alternative spliced cassette exons and alternative spliced cassette exons

First, since there are more non-alternative spliced (NAS) cassette exons than alternative spliced (AS) cassette exons, we equally divided the non-alternative spliced cassette exons into six parts and each part has similar number of non-alternative spliced cassette exons as the alternative spliced cassette exons. By combining the same alternative spliced cassette exons with different non-alternative spliced cassette exon parts, we compiled six datasets. Then, on each balanced dataset, we utilized mRMR+IFS approach to identify the important features for predicting alternative spliced cassette exons. The mRMR+IFS approach will be described in the following sections. Finally, the identified features on each datasets were combined.

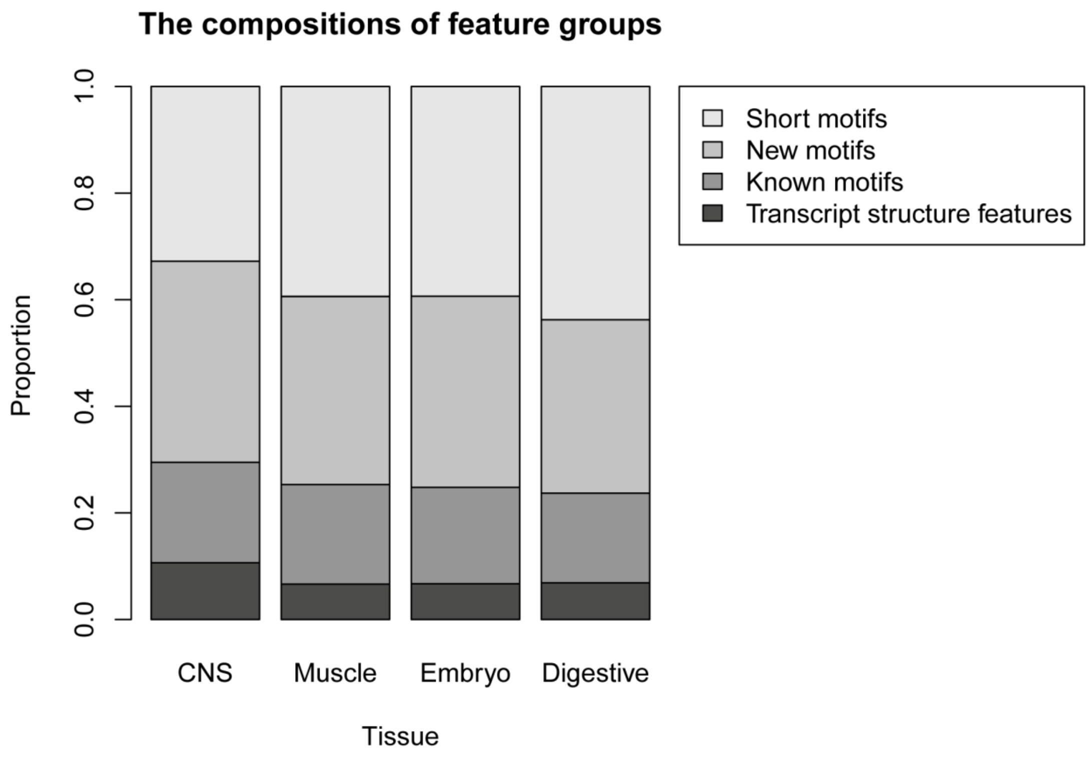

Figure S3 - The compositions of different feature groups in each tissue

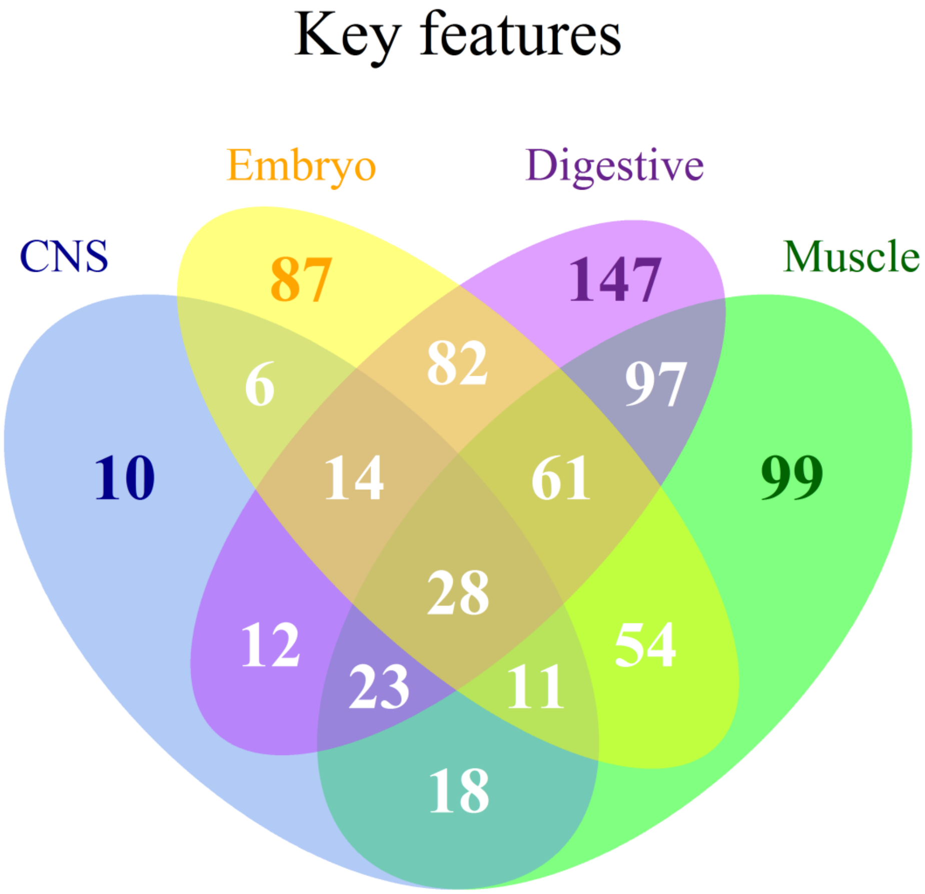

Figure S4 - Venn diagram for features identified across 4 tissue types

Both overlapped and tissue- specific features are shown.

Supplementary Tables

Table S1 - The number of non-alternative spliced and alternative spliced cassette exons

Tissue	Number of non-alternative spliced cassette exons	Number of alternative spliced cassette exons
CNS	3173	492
Muscle	3155	510
Embryo	3018	647
Digestive	2955	710

Table S2 - The 1,014 features of cassette exon and their annotations

**Feature Name**	**Label**	**Region**	**Type**
cis.ShortSeq.AC.C2I2	NucFreq.C2I2	I2(3')	short motifs
cis.Fox.v2.C2I2	Fox	I2(3')	known motifs
cis.Fox.v3.C2I2	Fox	I2(3')	known motifs
cis.Fox.v1.C2I2	Fox	I2(3')	known motifs
cis.Nova.NISS1	Nova	NISS1	known motifs
cis.Nova.NISS2	Nova	NISS2	known motifs
cis.Fox.v2.I2	Fox	I2(5')	known motifs
cis.Fox.v3.I2	Fox	I2(5')	known motifs
cis.GUrich.v1.I2	GUrich	I2(5')	known motifs
cis.GUrich.v2.I2	GUrich	I2(5')	known motifs
cis.GUrich.v3.I2	GUrich	I2(5')	known motifs
cis.GUrich.v4.I2	GUrich	I2(5')	known motifs
cis.Tra2.I2	Tra2	I2(5')	known motifs
cis.UGrich.v1.I2	UGrich	I2(5')	known motifs
cis.UGrich.v2.I2	UGrich	I2(5')	known motifs
cis.CUGBP.n2.I2	Cugbp	I2(5')	known motifs
cis.CUGBP.n3.I2	Cugbp	I2(5')	known motifs
cis.CELFlike.I2	Celf	I2(5')	known motifs
cis.Quak.I2	Quaking	I2(5')	known motifs
cis.MBNL.v1.I2	Mbnl	I2(5')	known motifs

cis.MBNL.v2.I2	Mbnl	I2(5')	known motifs
cis.MBNL.v3.I2	Mbnl	I2(5')	known motifs
cis.Nova.I2	Nova	I2(5')	known motifs
cis.Grich.I2	Grich	I2(5')	known motifs
cis.Trich.WithC.I2	Urich	I2(5')	known motifs
cis.Trich.WithG.I2	Urich	I2(5')	known motifs
cis.Trich.I2	Urich	I2(5')	known motifs
cis.Fox.v1.I2	Fox	I2(5')	known motifs
cis.PTB.v1.I2	(n)PTB	I2(5')	known motifs
cis.PTB.v2.I2	(n)PTB	I2(5')	known motifs
cis.PTB.v3.I2	(n)PTB	I2(5')	known motifs
cis.PTB.v4.I2	(n)PTB	I2(5')	known motifs
cis.Fox.v2.I1	Fox	I1(3')	known motifs
cis.Fox.v3.I1	Fox	I1(3')	known motifs
cis.GUrich.v1.I1	GUrich	I1(3')	known motifs
cis.GUrich.v2.I1	GUrich	I1(3')	known motifs
cis.GUrich.v3.I1	GUrich	I1(3')	known motifs
cis.GUrich.v4.I1	GUrich	I1(3')	known motifs
cis.Tra2.I1	Tra2	I1(3')	known motifs
cis.UGrich.v1.I1	UGrich	I1(3')	known motifs
cis.UGrich.v2.I1	UGrich	I1(3')	known motifs
cis.CUGBP.n2.I1	Cugbp	I1(3')	known motifs
cis.CUGBP.n3.I1	Cugbp	I1(3')	known motifs
cis.MBNL.v1.I1	Mbnl	I1(3')	known motifs
cis.MBNL.v2.I1	Mbnl	I1(3')	known motifs
cis.MBNL.v3.I1	Mbnl	I1(3')	known motifs
cis.Nova.I1	Nova	I1(3')	known motifs
cis.Sugnet.I1	Sugnet	I1(3')	known motifs
cis.Fox.v1.I1	Fox	I1(3')	known motifs
cis.PTB.v1.I1	(n)PTB	I1(3')	known motifs
cis.PTB.v2.I1	(n)PTB	I1(3')	known motifs
cis.PTB.v3.I1	(n)PTB	I1(3')	known motifs
cis.PTB.v4.I1	(n)PTB	I1(3')	known motifs
cisCons.Nova.AI2_C2I2	Nova	I2(5'),I2(3')	known motifs

cisCons.Nova.C1I1_AI1	Nova	I1(3'),I1(5')	known motifs
cisCons.Nova.NISE3	Nova	NISE3	known motifs
cisCons.CUGBP.n2.I1I2	Cugbp	I1(3'),I2(5')	known motifs
cisCons.CUGBP.n3.I1I2	Cugbp	I1(3'),I2(5')	known motifs
cisCons.Nova.I1I2	Nova	I1(3'),I2(5')	known motifs
cisCons.Fox.v1.I1I2	Fox	I1(3'),I2(5')	known motifs
cisCons.PTB.v1.I1I2	(n)PTB	I1(3'),I2(5')	known motifs
cisCons.PTB.v2.I1I2	(n)PTB	I1(3'),I2(5')	known motifs
cisCons.PTB.v3.I1I2	(n)PTB	I1(3'),I2(5')	known motifs
cisCons.PTB.v4.I1I2	(n)PTB	I1(3'),I2(5')	known motifs
cisCons.Nova.NESE1_NESS2	Nova	NESE1_NESS2	known motifs
cisCons.Fox.v2.C1I1	Fox	I1(5')	known motifs
cisCons.Fox.v3.C1I1	Fox	I1(5')	known motifs
cisCons.Fox.v1.C1I1	Fox	I1(5')	known motifs
cisCons.Nova.NISE2	Nova	NISE2	known motifs
cisCons.Nova.NISE1	Nova	NISE1	known motifs
cisCons.Nova.NISS1	Nova	NISS1	known motifs
cisCons.Fox.v2.C2I2	Fox	I2(3')	known motifs
cisCons.Fox.v3.C2I2	Fox	I2(3')	known motifs
cisCons.Fox.v1.C2I2	Fox	I2(3')	known motifs
cisCons.Nova.NISS2	Nova	NISS2	known motifs
cisCons.Fox.v2.I2	Fox	I2(5')	known motifs
cisCons.Fox.v3.I2	Fox	I2(5')	known motifs
cisCons.GUrich.v1.I2	GUrich	I2(5')	known motifs
cisCons.GUrich.v2.I2	GUrich	I2(5')	known motifs
cisCons.GUrich.v3.I2	GUrich	I2(5')	known motifs
cisCons.GUrich.v4.I2	GUrich	I2(5')	known motifs
cisCons.Tra2.I2	Tra2	I2(5')	known motifs
cisCons.UGrich.v1.I2	UGrich	I2(5')	known motifs
cisCons.UGrich.v2.I2	UGrich	I2(5')	known motifs
cisCons.CUGBP.n2.I2	Cugbp	I2(5')	known motifs
cisCons.CUGBP.n3.I2	Cugbp	I2(5')	known motifs
cisCons.CELFlike.I2	Celf	I2(5')	known motifs
cisCons.Quak.I2	Quaking	I2(5')	known motifs

cisCons.MBNL.v1.I2	Mbnl	I2(5')	known motifs
cisCons.MBNL.v2.I2	Mbnl	I2(5')	known motifs
cisCons.MBNL.v3.I2	Mbnl	I2(5')	known motifs
cisCons.Nova.I2	Nova	I2(5')	known motifs
cisCons.Grich.I2	Grich	I2(5')	known motifs
cisCons.Trich.WithC.I2	Urich	I2(5')	known motifs
cisCons.Trich.WithG.I2	Urich	I2(5')	known motifs
cisCons.Trich.I2	Urich	I2(5')	known motifs
cisCons.Fox.v1.I2	Fox	I2(5')	known motifs
cisCons.PTB.v1.I2	(n)PTB	I2(5')	known motifs
cisCons.PTB.v2.I2	(n)PTB	I2(5')	known motifs
cisCons.PTB.v3.I2	(n)PTB	I2(5')	known motifs
cisCons.PTB.v4.I2	(n)PTB	I2(5')	known motifs
cisCons.Fox.v2.I1	Fox	I1(3')	known motifs
cisCons.Fox.v3.I1	Fox	I1(3')	known motifs
cisCons.GUrich.v1.I1	GUrich	I1(3')	known motifs
cisCons.GUrich.v2.I1	GUrich	I1(3')	known motifs
cisCons.GUrich.v3.I1	GUrich	I1(3')	known motifs
cisCons.GUrich.v4.I1	GUrich	I1(3')	known motifs
cisCons.Tra2.I1	Tra2	I1(3')	known motifs
cisCons.UGrich.v1.I1	UGrich	I1(3')	known motifs
cisCons.UGrich.v2.I1	UGrich	I1(3')	known motifs
cisCons.CUGBP.n2.I1	Cugbp	I1(3')	known motifs
cisCons.CUGBP.n3.I1	Cugbp	I1(3')	known motifs
cisCons.MBNL.v1.I1	Mbnl	I1(3')	known motifs
cisCons.MBNL.v2.I1	Mbnl	I1(3')	known motifs
cisCons.MBNL.v3.I1	Mbnl	I1(3')	known motifs
cisCons.Nova.I1	Nova	I1(3')	known motifs
cisCons.Sugnet.I1	Sugnet	I1(3')	known motifs
cisCons.Fox.v1.I1	Fox	I1(3')	known motifs
cisCons.PTB.v1.I1	(n)PTB	I1(3')	known motifs
cisCons.PTB.v2.I1	(n)PTB	I1(3')	known motifs
cisCons.PTB.v3.I1	(n)PTB	I1(3')	known motifs
cisCons.PTB.v4.I1	(n)PTB	I1(3')	known motifs

cis.PSSM.SC35.C1	SC35	C1	known motifs
cis.PSSM.SF2_ASF.C1	SF2_ASF	C1	known motifs
cis.PSSM.SRp40.C1	SRp40	C1	known motifs
cis.PSSM.SRp55.C1	SRp55	C1	known motifs
cis.PSSM.SC35.A	SC35	A	known motifs
cis.PSSM.SF2_ASF.A	SF2_ASF	A	known motifs
cis.PSSM.SRp40.A	SRp40	A	known motifs
cis.PSSM.SRp55.A	SRp55	A	known motifs
cis.PSSM.SC35.C2	SC35	C2	known motifs
cis.PSSM.SF2_ASF.C2	SF2_ASF	C2	known motifs
cis.PSSM.SRp40.C2	SRp40	C2	known motifs
cis.PSSM.SRp55.C2	SRp55	C2	known motifs
cis.PSSM.SC35.I1	SC35	I1(3')	known motifs
cis.PSSM.SF2_ASF.I1	SF2_ASF	I1(3')	known motifs
cis.PSSM.SRp40.I1	SRp40	I1(3')	known motifs
cis.PSSM.SRp55.I1	SRp55	I1(3')	known motifs
cis.PSSM.SC35.I2	SC35	I2(5')	known motifs
cis.PSSM.SF2_ASF.I2	SF2_ASF	I2(5')	known motifs
cis.PSSM.SRp40.I2	SRp40	I2(5')	known motifs
cis.PSSM.SRp55.I2	SRp55	I2(5')	known motifs
cis.NovaClust.NESE1_NESS2	Nova	NESE1_NESS2	known motifs
cis.NovaClust.NESS1	Nova	NESS1	known motifs
cis.NovaClust.NISE1	Nova	NISE1	known motifs
cis.NovaClust.NISE2	Nova	NISE2	known motifs
cis.NovaClust.NISE3	Nova	NISE3	known motifs
cis.NovaClust.NISS1	Nova	NISS1	known motifs
cis.NovaClust.NISS2	Nova	NISS2	known motifs
cis.Nova.NESS1	Nova	NESS1	known motifs
cis.Nova.AI2_C2I2	Nova	I2(5'),I2(3')	known motifs
cis.Nova.C1I1_AI1	Nova	I1(3'),I1(5')	known motifs
cis.Nova.C1	Nova	C1	known motifs
cis.Nova.NISE3	Nova	NISE3	known motifs
cis.CUGBP.n2.I1I2	Cugbp	I1(3'),I2(5')	known motifs
cis.CUGBP.n3.I1I2	Cugbp	I1(3'),I2(5')	known motifs

cis.Nova.I1I2	Nova	I1(3'),I2(5')	known motifs
cis.Fox.v1.I1I2	Fox	I1(3'),I2(5')	known motifs
cis.PTB.v1.I1I2	(n)PTB	I1(3'),I2(5')	known motifs
cis.PTB.v2.I1I2	(n)PTB	I1(3'),I2(5')	known motifs
cis.PTB.v3.I1I2	(n)PTB	I1(3'),I2(5')	known motifs
cis.PTB.v4.I1I2	(n)PTB	I1(3'),I2(5')	known motifs
cis.Nova.NESE1_NESS2	Nova	NESE1_NESS2	known motifs
cis.Nova.C2	Nova	C2	known motifs
cis.Fox.v2.C1I1	Fox	I1(5')	known motifs
cis.Fox.v3.C1I1	Fox	I1(5')	known motifs
cis.Fox.v1.C1I1	Fox	I1(5')	known motifs
cis.Tra2.A	Tra2	A	known motifs
cis.Nova.A	Nova	A	known motifs
cis.Nova.C1_A	Nova	C1_A	known motifs
cis.Nova.NISE2	Nova	NISE2	known motifs
cis.Nova.NISE1	Nova	NISE1	known motifs
cisCons.YeoClustDown.1.GTAAC.I2	Motif.I2	I2(5')	new motifs
cisCons.YeoClustDown.2.AAGTGT.I2	Motif.I2	I2(5')	new motifs
cisCons.YeoClustDown.3.GTTTGT.I2	Motif.I2	I2(5')	new motifs
cisCons.YeoClustDown.4.ATTAACA.I2	Motif.I2	I2(5')	new motifs
cisCons.YeoClustDown.5.TGAAG.I2	Motif.I2	I2(5')	new motifs
cisCons.YeoClustDown.6.TAACC.I2	Motif.I2	I2(5')	new motifs
cisCons.YeoClustDown.7.TTGAAAT.I2	Motif.I2	I2(5')	new motifs
cisCons.YeoClustDown.8.AATTG.I2	Motif.I2	I2(5')	new motifs
cisCons.YeoClustDown.9.CTGCT.I2	Motif.I2	I2(5')	new motifs
cisCons.YeoClustDown.10.TTTATG.I2	Motif.I2	I2(5')	new motifs
cisCons.YeoClustDown.11.TGATAAA.I2	Motif.I2	I2(5')	new motifs
cisCons.YeoClustDown.12.ATGTTT.I2	Motif.I2	I2(5')	new motifs
cisCons.YeoClustDown.13.TTTCCAA.I2	Motif.I2	I2(5')	new motifs
cisCons.YeoClustDown.14.AAGTC.I2	Motif.I2	I2(5')	new motifs
cisCons.YeoClustDown.15.AAAGA.I2	Motif.I2	I2(5')	new motifs
cisCons.YeoClustDown.16.GTACGT.I2	Motif.I2	I2(5')	new motifs
cisCons.YeoClustDown.17.GTTAAA.I2	Motif.I2	I2(5')	new motifs
cisCons.YeoClustDown.18.GAGCTG.I2	Motif.I2	I2(5')	new motifs

cisCons.YeoClustDown.19.TCATTTT.I2	Motif.I2	I2(5')	new motifs
cisCons.YeoClustDown.20.TGCATG.I2	Fox	I2(5')	new motifs
cisCons.YeoClustDown.21.TTCTT.I2	(n)PTB	I2(5')	new motifs
cisCons.YeoClustDown.22.TTTATC.I2	Motif.I2	I2(5')	new motifs
cisCons.YeoClustDown.23.ACATTT.I2	Motif.I2	I2(5')	new motifs
cisCons.YeoClustDown.24.TGCCAGC.I2	Motif.I2	I2(5')	new motifs
cisCons.YeoClustDown.25.ATAATT.I2	Motif.I2	I2(5')	new motifs
cisCons.YeoClustDown.26.GTAGG.I2	Motif.I2	I2(5')	new motifs
cisCons.YeoClustDown.27.GTATCCT.I2	Motif.I2	I2(5')	new motifs
cisCons.YeoClustDown.28.CATTTG.I2	Motif.I2	I2(5')	new motifs
cisCons.YeoClustDown.29.ACTAAC.I2	Quaking	I2(5')	new motifs
cisCons.YeoClustDown.30.TTTCAG.I2	Motif.I2	I2(5')	new motifs
cisCons.YeoClustDown.31.AATTGA.I2	Motif.I2	I2(5')	new motifs
cisCons.YeoClustDown.32.TTAGCA.I2	Motif.I2	I2(5')	new motifs
cisCons.YeoClustDown.33.CAAAT.I2	Motif.I2	I2(5')	new motifs
cisCons.YeoClustDown.34.TAATG.I2	Motif.I2	I2(5')	new motifs
cisCons.YeoClustDown.35.TTTTGAT.I2	Motif.I2	I2(5')	new motifs
cisCons.YeoClustDown.36.AGAAAT.I2	Motif.I2	I2(5')	new motifs
cisCons.YeoClustDown.37.TTTCTA.I2	(n)PTB	I2(5')	new motifs
cisCons.YeoClustDown.38.TATTTC.I2	Motif.I2	I2(5')	new motifs
cisCons.YeoClustDown.39.TAACT.I2	Motif.I2	I2(5')	new motifs
cisCons.YeoClustDown.40.TGAGG.I2	Motif.I2	I2(5')	new motifs
cisCons.YeoClustDown.41.TAAAAT.I2	Motif.I2	I2(5')	new motifs
cisCons.YeoClustDown.42.TTTATA.I2	Motif.I2	I2(5')	new motifs
cisCons.YeoClustDown.43.TATCCT.I2	Motif.I2	I2(5')	new motifs
cisCons.YeoClustDown.44.GTTAGT.I2	Motif.I2	I2(5')	new motifs
cisCons.YeoClustDown.45.TTTACAG.I2	Motif.I2	I2(5')	new motifs
cisCons.YeoClustDown.46.TATTTG.I2	Motif.I2	I2(5')	new motifs
cisCons.YeoClustDown.47.GTACTGT.I2	Motif.I2	I2(5')	new motifs
cisCons.YeoClustDown.48.TTAAG.I2	Motif.I2	I2(5')	new motifs
cisCons.YeoClustDown.49.CATAAA.I2	Motif.I2	I2(5')	new motifs
cisCons.YeoClustDown.50.GCATG.I2	Fox	I2(5')	new motifs
cisCons.YeoClustDown.51.TGATTA.I2	Motif.I2	I2(5')	new motifs
cisCons.YeoClustDown.52.TTTTAAA.I2	Motif.I2	I2(5')	new motifs

cisCons.YeoClustDown.53.CTGACT.I2	Motif.I2	I2(5')	new motifs
cisCons.YeoClustDown.54.ACTAAT.I2	Motif.I2	I2(5')	new motifs
cisCons.YeoClustDown.55.GAGTA.I2	Motif.I2	I2(5')	new motifs
cisCons.YeoClustDown.56.TCTTAA.I2	(n)PTB	I2(5')	new motifs
cisCons.YeoClustDown.57.TTGGTT.I2	Motif.I2	I2(5')	new motifs
cisCons.YeoClustDown.58.ATATTT.I2	Motif.I2	I2(5')	new motifs
cisCons.YeoClustDown.59.AGAGCCA.I2	Motif.I2	I2(5')	new motifs
cisCons.YeoClustDown.60.TCTTT.I2	(n)PTB	I2(5')	new motifs
cisCons.YeoClustDown.61.AGTTTT.I2	Motif.I2	I2(5')	new motifs
cisCons.YeoClustDown.62.GTATTT.I2	Motif.I2	I2(5')	new motifs
cisCons.YeoClustDown.63.TCAGA.I2	Motif.I2	I2(5')	new motifs
cisCons.YeoClustDown.64.TAAGT.I2	Motif.I2	I2(5')	new motifs
cisCons.YeoClustDown.65.AAGCA.I2	Motif.I2	I2(5')	new motifs
cisCons.YeoClustDown.66.TTCACAG.I2	Motif.I2	I2(5')	new motifs
cisCons.YeoClustDown.67.AGTAA.I2	Motif.I2	I2(5')	new motifs
cisCons.YeoClustDown.68.TCTGG.I2	(n)PTB	I2(5')	new motifs
cisCons.YeoClustDown.69.AGCTTT.I2	Motif.I2	I2(5')	new motifs
cisCons.YeoClustDown.70.TGATTTG.I2	Motif.I2	I2(5')	new motifs
cisCons.YeoClustDown.71.TTTTGC.I2	Motif.I2	I2(5')	new motifs
cisCons.YeoClustDown.72.TAGAAA.I2	Motif.I2	I2(5')	new motifs
cisCons.YeoClustDown.73.GTGAG.I2	Motif.I2	I2(5')	new motifs
cisCons.YeoClustDown.74.TTCTGT.I2	(n)PTB	I2(5')	new motifs
cisCons.YeoClustDown.75.GTAAG.I2	Motif.I2	I2(5')	new motifs
cisCons.YeoClustDown.76.ATGAAA.I2	Motif.I2	I2(5')	new motifs
cisCons.YeoClustDown.77.AGAAAA.I2	Motif.I2	I2(5')	new motifs
cisCons.YeoClustDown.78.TGAGC.I2	Motif.I2	I2(5')	new motifs
cisCons.YeoClustDown.79.TGGCTT.I2	Motif.I2	I2(5')	new motifs
cisCons.YeoClustDown.80.TTAATCT.I2	(n)PTB	I2(5')	new motifs
cisCons.YeoClustDown.81.AATTAT.I2	Motif.I2	I2(5')	new motifs
cisCons.YeoClustDown.82.TGGAAAT.I2	Motif.I2	I2(5')	new motifs
cisCons.YeoClustDown.83.CCACAG.I2	Motif.I2	I2(5')	new motifs
cisCons.YeoClustDown.84.AAATGA.I2	Motif.I2	I2(5')	new motifs
cisCons.YeoClustDown.85.GCAAGT.I2	Motif.I2	I2(5')	new motifs
cisCons.YeoClustDown.86.GTAAAA.I2	Motif.I2	I2(5')	new motifs

cisCons.YeoClustDown.87.GTCTG.I2	(n)PTB	I2(5')	new motifs
cisCons.YeoClustDown.88.AAATGT.I2	Motif.I2	I2(5')	new motifs
cisCons.YeoClustDown.89.TGCAT.I2	Fox	I2(5')	new motifs
cisCons.YeoClustDown.90.GAGAAA.I2	Motif.I2	I2(5')	new motifs
cisCons.YeoClustDown.91.TTAGA.I2	Motif.I2	I2(5')	new motifs
cisCons.YeoClustDown.92.TTTATAA.I2	Motif.I2	I2(5')	new motifs
cisCons.YeoClustDown.93.GTTTT.I2	Motif.I2	I2(5')	new motifs
cisCons.YeoClustDown.94.GCTTGGC.I2	Motif.I2	I2(5')	new motifs
cisCons.YeoClustDown.95.TAAGC.I2	Motif.I2	I2(5')	new motifs
cisCons.YeoClustDown.96.GTATG.I2	Motif.I2	I2(5')	new motifs
cisCons.YeoClustDown.97.AAATT.I2	Motif.I2	I2(5')	new motifs
cisCons.YeoClustDown.98.GTAAT.I2	Motif.I2	I2(5')	new motifs
cisCons.YeoClustDown.99.TTCTCT.I2	(n)PTB	I2(5')	new motifs
cisCons.YeoClustDown.100.TGAGAA.I2	Motif.I2	I2(5')	new motifs
cisCons.YeoClustDown.101.TTAGTT.I2	Motif.I2	I2(5')	new motifs
cisCons.YeoClustDown.102.TAAGG.I2	Motif.I2	I2(5')	new motifs
cisCons.YeoClustDown.103.TGTTTAA.I2	Motif.I2	I2(5')	new motifs
cisCons.YeoClustDown.104.GTCAGT.I2	Motif.I2	I2(5')	new motifs
cisCons.YeoClustDown.105.AGAATT.I2	Motif.I2	I2(5')	new motifs
cisCons.YeoClustDown.106.TAAATG.I2	Motif.I2	I2(5')	new motifs
cisCons.YeoClustDown.107.AATTCA.I2	Motif.I2	I2(5')	new motifs
cisCons.YeoClustDown.108.TCCTTT.I2	Motif.I2	I2(5')	new motifs
cisCons.YeoClustDown.109.TAAGA.I2	Motif.I2	I2(5')	new motifs
cisCons.YeoClustDown.110.AAATCA.I2	Motif.I2	I2(5')	new motifs
cisCons.YeoClustDown.111.TAATTTG.I2	Motif.I2	I2(5')	new motifs
cisCons.YeoClustDown.112.GAAATA.I2	Motif.I2	I2(5')	new motifs
cisCons.YeoClustDown.113.TGGTTT.I2	Motif.I2	I2(5')	new motifs
cisCons.YeoClustDown.114.TGTTAA.I2	Motif.I2	I2(5')	new motifs
cisCons.YeoClustDown.115.TGTCT.I2	(n)PTB	I2(5')	new motifs
cisCons.YeoClustDown.116.GTTGGT.I2	Motif.I2	I2(5')	new motifs
cisCons.YeoClustDown.117.TGAATT.I2	Motif.I2	I2(5')	new motifs
cisCons.YeoClustDown.118.AATTTA.I2	Motif.I2	I2(5')	new motifs
cisCons.YeoClustDown.119.TATGT.I2	Motif.I2	I2(5')	new motifs
cisCons.YeoClustDown.120.GCATTT.I2	Motif.I2	I2(5')	new motifs

cisCons.YeoClustDown.121.AAGTA.I2	Motif.I2	I2(5')	new motifs
cisCons.YeoClustDown.122.GCTTCT.I2	(n)PTB	I2(5')	new motifs
cisCons.YeoClustDown.123.TTCTAA.I2	(n)PTB	I2(5')	new motifs
cisCons.YeoClustDown.124.GTTTCT.I2	(n)PTB	I2(5')	new motifs
cisCons.YeoClustDown.125.AGATTT.I2	Motif.I2	I2(5')	new motifs
cisCons.YeoClustDown.126.GAAAAT.I2	Motif.I2	I2(5')	new motifs
cisCons.YeoClustDown.127.TGCTAA.I2	Motif.I2	I2(5')	new motifs
cisCons.YeoClustDown.128.AAGCT.I2	Motif.I2	I2(5')	new motifs
cisCons.YeoClustDown.129.CTTTGCT.I2	Motif.I2	I2(5')	new motifs
cisCons.YeoClustDown.130.TCTGA.I2	(n)PTB	I2(5')	new motifs
cisCons.YeoClustDown.131.TTTCTC.I2	(n)PTB	I2(5')	new motifs
cisCons.YeoClustDown.132.TTTATTC.I2	Motif.I2	I2(5')	new motifs
cisCons.YeoClustDown.133.TTTGCC.I2	Motif.I2	I2(5')	new motifs
cisCons.YeoClustDown.134.TGAAAG.I2	Motif.I2	I2(5')	new motifs
cisCons.YeoClustDown.135.TGTTCT.I2	(n)PTB	I2(5')	new motifs
cisCons.YeoClustDown.136.CTTTT.I2	Motif.I2	I2(5')	new motifs
cisCons.YeoClustDown.137.TTTTCTG.I2	(n)PTB	I2(5')	new motifs
cisCons.YeoClustDown.138.TGAGT.I2	Motif.I2	I2(5')	new motifs
cisCons.YeoClustDown.139.TTGCAG.I2	Motif.I2	I2(5')	new motifs
cisCons.YeoClustDown.140.TAATA.I2	Motif.I2	I2(5')	new motifs
cisCons.YeoClustDown.141.AGTAT.I2	Motif.I2	I2(5')	new motifs
cisCons.YeoClustDown.142.ATTCT.I2	(n)PTB	I2(5')	new motifs
cisCons.YeoClustDown.143.TGCCTTT.I2	Motif.I2	I2(5')	new motifs
cisCons.YeoClustDown.144.ATCAAA.I2	Motif.I2	I2(5')	new motifs
cisCons.YeoClustDown.145.GAGTG.I2	Motif.I2	I2(5')	new motifs
cisCons.YeoClustDown.146.TAGGT.I2	Motif.I2	I2(5')	new motifs
cisCons.YeoClustDown.147.CTTTA.I2	Motif.I2	I2(5')	new motifs
cisCons.YeoClustDown.148.TTTAG.I2	Motif.I2	I2(5')	new motifs
cisCons.YeoClustDown.149.TGATTTT.I2	Motif.I2	I2(5')	new motifs
cisCons.YeoClustDown.150.TTTCAT.I2	Motif.I2	I2(5')	new motifs
cisCons.YeoClustDown.151.CTTTCA.I2	Motif.I2	I2(5')	new motifs
cisCons.YeoClustDown.152.AAGAT.I2	Motif.I2	I2(5')	new motifs
cisCons.YeoClustDown.153.TGCTT.I2	Motif.I2	I2(5')	new motifs
cisCons.YeoClustDown.154.GTGGGT.I2	Motif.I2	I2(5')	new motifs

cisCons.YeoClustDown.155.GTAAAG.I2	Motif.I2	I2(5')	new motifs
cisCons.YeoClustDown.156.CTGAA.I2	Motif.I2	I2(5')	new motifs
cisCons.YeoClustDown.157.TCTGC.I2	(n)PTB	I2(5')	new motifs
cisCons.YeoClustDown.158.CTAAA.I2	Motif.I2	I2(5')	new motifs
cisCons.YeoClustUp.1.GTTTGT.I1	Motif.I1	I1(3')	new motifs
cisCons.YeoClustUp.2.TCTCC.I1	(n)PTB	I1(3')	new motifs
cisCons.YeoClustUp.3.GATTTT.I1	Motif.I1	I1(3')	new motifs
cisCons.YeoClustUp.4.TTTTTC.I1	Motif.I1	I1(3')	new motifs
cisCons.YeoClustUp.5.TAACC.I1	Motif.I1	I1(3')	new motifs
cisCons.YeoClustUp.6.TTGAAAT.I1	Motif.I1	I1(3')	new motifs
cisCons.YeoClustUp.7.AAGCCA.I1	Motif.I1	I1(3')	new motifs
cisCons.YeoClustUp.8.AATTG.I1	Motif.I1	I1(3')	new motifs
cisCons.YeoClustUp.9.CTGCT.I1	Motif.I1	I1(3')	new motifs
cisCons.YeoClustUp.10.TTTATG.I1	Motif.I1	I1(3')	new motifs
cisCons.YeoClustUp.11.TTCACA.I1	Motif.I1	I1(3')	new motifs
cisCons.YeoClustUp.12.TGATAA.I1	Motif.I1	I1(3')	new motifs
cisCons.YeoClustUp.13.ATGTTT.I1	Motif.I1	I1(3')	new motifs
cisCons.YeoClustUp.14.TCCAG.I1	Motif.I1	I1(3')	new motifs
cisCons.YeoClustUp.15.TTTCCAA.I1	Motif.I1	I1(3')	new motifs
cisCons.YeoClustUp.16.TTATTTC.I1	Motif.I1	I1(3')	new motifs
cisCons.YeoClustUp.17.TGTGTT.I1	Motif.I1	I1(3')	new motifs
cisCons.YeoClustUp.18.TCTTG.I1	(n)PTB	I1(3')	new motifs
cisCons.YeoClustUp.19.TTGTAA.I1	Motif.I1	I1(3')	new motifs
cisCons.YeoClustUp.20.CTTGAC.I1	Motif.I1	I1(3')	new motifs
cisCons.YeoClustUp.21.TTAAAAC.I1	Motif.I1	I1(3')	new motifs
cisCons.YeoClustUp.22.CTAAC.I1	Quaking	I1(3')	new motifs
cisCons.YeoClustUp.23.AAAGCT.I1	Motif.I1	I1(3')	new motifs
cisCons.YeoClustUp.24.TCTTC.I1	(n)PTB	I1(3')	new motifs
cisCons.YeoClustUp.25.TGCATG.I1	Fox	I1(3')	new motifs
cisCons.YeoClustUp.26.TTCTT.I1	(n)PTB	I1(3')	new motifs
cisCons.YeoClustUp.27.ACATTT.I1	Motif.I1	I1(3')	new motifs
cisCons.YeoClustUp.28.TTTATC.I1	Motif.I1	I1(3')	new motifs
cisCons.YeoClustUp.29.ATTTTCT.I1	(n)PTB	I1(3')	new motifs
cisCons.YeoClustUp.30.GCTGACC.I1	Motif.I1	I1(3')	new motifs

cisCons.YeoClustUp.31.ATAATT.I1	Motif.I1	I1(3')	new motifs
cisCons.YeoClustUp.32.AACAG.I1	Motif.I1	I1(3')	new motifs
cisCons.YeoClustUp.33.CATTTG.I1	Motif.I1	I1(3')	new motifs
cisCons.YeoClustUp.34.TTTCAG.I1	Motif.I1	I1(3')	new motifs
cisCons.YeoClustUp.35.TTAGCA.I1	Motif.I1	I1(3')	new motifs
cisCons.YeoClustUp.36.TTGCCT.I1	Motif.I1	I1(3')	new motifs
cisCons.YeoClustUp.37.CAAAT.I1	Motif.I1	I1(3')	new motifs
cisCons.YeoClustUp.38.TTTAAC.I1	Motif.I1	I1(3')	new motifs
cisCons.YeoClustUp.39.TAATG.I1	Motif.I1	I1(3')	new motifs
cisCons.YeoClustUp.40.AGAAAT.I1	Motif.I1	I1(3')	new motifs
cisCons.YeoClustUp.41.TTTTGAT.I1	Motif.I1	I1(3')	new motifs
cisCons.YeoClustUp.42.TAACT.I1	Motif.I1	I1(3')	new motifs
cisCons.YeoClustUp.43.TAAAAT.I1	Motif.I1	I1(3')	new motifs
cisCons.YeoClustUp.44.AATTACA.I1	Motif.I1	I1(3')	new motifs
cisCons.YeoClustUp.45.TTCAAAA.I1	Motif.I1	I1(3')	new motifs
cisCons.YeoClustUp.46.TTTATA.I1	Motif.I1	I1(3')	new motifs
cisCons.YeoClustUp.47.CTTGTC.I1	Motif.I1	I1(3')	new motifs
cisCons.YeoClustUp.48.TTTACAG.I1	Motif.I1	I1(3')	new motifs
cisCons.YeoClustUp.49.TGGATTT.I1	Motif.I1	I1(3')	new motifs
cisCons.YeoClustUp.50.TTGCATT.I1	Fox	I1(3')	new motifs
cisCons.YeoClustUp.51.TTAAG.I1	Motif.I1	I1(3')	new motifs
cisCons.YeoClustUp.52.TTGGT.I1	Motif.I1	I1(3')	new motifs
cisCons.YeoClustUp.53.CATAAA.I1	Motif.I1	I1(3')	new motifs
cisCons.YeoClustUp.54.TGATTA.I1	Motif.I1	I1(3')	new motifs
cisCons.YeoClustUp.55.GCTTTGC.I1	Motif.I1	I1(3')	new motifs
cisCons.YeoClustUp.56.ATTAG.I1	Motif.I1	I1(3')	new motifs
cisCons.YeoClustUp.57.TTTTAAA.I1	Motif.I1	I1(3')	new motifs
cisCons.YeoClustUp.58.ACTAAT.I1	Motif.I1	I1(3')	new motifs
cisCons.YeoClustUp.59.CTGACT.I1	Motif.I1	I1(3')	new motifs
cisCons.YeoClustUp.60.ATATTT.I1	Motif.I1	I1(3')	new motifs
cisCons.YeoClustUp.61.CATTTA.I1	Motif.I1	I1(3')	new motifs
cisCons.YeoClustUp.62.AAATCT.I1	(n)PTB	I1(3')	new motifs
cisCons.YeoClustUp.63.TTTTGGC.I1	Motif.I1	I1(3')	new motifs
cisCons.YeoClustUp.64.TCTTT.I1	(n)PTB	I1(3')	new motifs

cisCons.YeoClustUp.65.TTATTGA.I1	Motif.I1	I1(3')	new motifs
cisCons.YeoClustUp.66.TCAGA.I1	Motif.I1	I1(3')	new motifs
cisCons.YeoClustUp.67.TAAGT.I1	Motif.I1	I1(3')	new motifs
cisCons.YeoClustUp.68.CTCTG.I1	(n)PTB	I1(3')	new motifs
cisCons.YeoClustUp.69.TCTGG.I1	(n)PTB	I1(3')	new motifs
cisCons.YeoClustUp.70.AATTC.I1	Motif.I1	I1(3')	new motifs
cisCons.YeoClustUp.71.TTTTCC.I1	Motif.I1	I1(3')	new motifs
cisCons.YeoClustUp.72.TTTTGC.I1	Motif.I1	I1(3')	new motifs
cisCons.YeoClustUp.73.GTGAG.I1	Motif.I1	I1(3')	new motifs
cisCons.YeoClustUp.74.TCCATTT.I1	Motif.I1	I1(3')	new motifs
cisCons.YeoClustUp.75.AATTTT.I1	Motif.I1	I1(3')	new motifs
cisCons.YeoClustUp.76.CTTGATT.I1	Motif.I1	I1(3')	new motifs
cisCons.YeoClustUp.77.GTAAG.I1	Motif.I1	I1(3')	new motifs
cisCons.YeoClustUp.78.ATGAAA.I1	Motif.I1	I1(3')	new motifs
cisCons.YeoClustUp.79.AGAAAA.I1	Motif.I1	I1(3')	new motifs
cisCons.YeoClustUp.80.TGGCTT.I1	Motif.I1	I1(3')	new motifs
cisCons.YeoClustUp.81.CTCAG.I1	(n)PTB	I1(3')	new motifs
cisCons.YeoClustUp.82.TGGAAAT.I1	Motif.I1	I1(3')	new motifs
cisCons.YeoClustUp.83.AATTAT.I1	Motif.I1	I1(3')	new motifs
cisCons.YeoClustUp.84.AATAAT.I1	Motif.I1	I1(3')	new motifs
cisCons.YeoClustUp.85.TCCTAG.I1	Motif.I1	I1(3')	new motifs
cisCons.YeoClustUp.86.CCACAG.I1	Motif.I1	I1(3')	new motifs
cisCons.YeoClustUp.87.TCATTTC.I1	Motif.I1	I1(3')	new motifs
cisCons.YeoClustUp.88.AAAGCA.I1	Motif.I1	I1(3')	new motifs
cisCons.YeoClustUp.89.AAATGA.I1	Motif.I1	I1(3')	new motifs
cisCons.YeoClustUp.90.TTTATAG.I1	Motif.I1	I1(3')	new motifs
cisCons.YeoClustUp.91.ATTAAAT.I1	Motif.I1	I1(3')	new motifs
cisCons.YeoClustUp.92.CCTGCAG.I1	Motif.I1	I1(3')	new motifs
cisCons.YeoClustUp.93.TTACAG.I1	Motif.I1	I1(3')	new motifs
cisCons.YeoClustUp.94.AAATGT.I1	Motif.I1	I1(3')	new motifs
cisCons.YeoClustUp.95.TGCAT.I1	Fox	I1(3')	new motifs
cisCons.YeoClustUp.96.CTTCT.I1	(n)PTB	I1(3')	new motifs
cisCons.YeoClustUp.97.TTAGAA.I1	Motif.I1	I1(3')	new motifs
cisCons.YeoClustUp.98.TGTTTC.I1	Motif.I1	I1(3')	new motifs

cisCons.YeoClustUp.99.TTTAC.I1	Motif.I1	I1(3')	new motifs
cisCons.YeoClustUp.100.GTTTT.I1	Motif.I1	I1(3')	new motifs
cisCons.YeoClustUp.101.CTTCCA.I1	Motif.I1	I1(3')	new motifs
cisCons.YeoClustUp.102.TTCTAG.I1	(n)PTB	I1(3')	new motifs
cisCons.YeoClustUp.103.AAATT.I1	Motif.I1	I1(3')	new motifs
cisCons.YeoClustUp.104.TTAAAC.I1	Motif.I1	I1(3')	new motifs
cisCons.YeoClustUp.105.TGAGAA.I1	Motif.I1	I1(3')	new motifs
cisCons.YeoClustUp.106.TTTGTAG.I1	Motif.I1	I1(3')	new motifs
cisCons.YeoClustUp.107.GTCAGT.I1	Motif.I1	I1(3')	new motifs
cisCons.YeoClustUp.108.TAAGA.I1	Motif.I1	I1(3')	new motifs
cisCons.YeoClustUp.109.AAATCA.I1	Motif.I1	I1(3')	new motifs
cisCons.YeoClustUp.110.TGTTGA.I1	Motif.I1	I1(3')	new motifs
cisCons.YeoClustUp.111.CTTGC.I1	Motif.I1	I1(3')	new motifs
cisCons.YeoClustUp.112.TAATTTG.I1	Motif.I1	I1(3')	new motifs
cisCons.YeoClustUp.113.CCTCT.I1	(n)PTB	I1(3')	new motifs
cisCons.YeoClustUp.114.TGGTTT.I1	Motif.I1	I1(3')	new motifs
cisCons.YeoClustUp.115.TGATTTC.I1	Motif.I1	I1(3')	new motifs
cisCons.YeoClustUp.116.TGTTAA.I1	Motif.I1	I1(3')	new motifs
cisCons.YeoClustUp.117.TGTGTC.I1	Motif.I1	I1(3')	new motifs
cisCons.YeoClustUp.118.TCTCT.I1	(n)PTB	I1(3')	new motifs
cisCons.YeoClustUp.119.TTAACA.I1	Motif.I1	I1(3')	new motifs
cisCons.YeoClustUp.120.TTTGGT.I1	Motif.I1	I1(3')	new motifs
cisCons.YeoClustUp.121.TGTCT.I1	(n)PTB	I1(3')	new motifs
cisCons.YeoClustUp.122.TTCCTT.I1	Motif.I1	I1(3')	new motifs
cisCons.YeoClustUp.123.TGAATT.I1	Motif.I1	I1(3')	new motifs
cisCons.YeoClustUp.124.AATTTA.I1	Motif.I1	I1(3')	new motifs
cisCons.YeoClustUp.125.GTTTCT.I1	(n)PTB	I1(3')	new motifs
cisCons.YeoClustUp.126.TGCTAA.I1	Motif.I1	I1(3')	new motifs
cisCons.YeoClustUp.127.AGATTT.I1	Motif.I1	I1(3')	new motifs
cisCons.YeoClustUp.128.GAAAAT.I1	Motif.I1	I1(3')	new motifs
cisCons.YeoClustUp.129.GTTTAAT.I1	Motif.I1	I1(3')	new motifs
cisCons.YeoClustUp.130.TTTGACT.I1	Motif.I1	I1(3')	new motifs
cisCons.YeoClustUp.131.TCTGA.I1	(n)PTB	I1(3')	new motifs
cisCons.YeoClustUp.132.TCTGTT.I1	(n)PTB	I1(3')	new motifs

cisCons.YeoClustUp.133.TTTATTC.I1	Motif.I1	I1(3')	new motifs
cisCons.YeoClustUp.134.TGAAAG.I1	Motif.I1	I1(3')	new motifs
cisCons.YeoClustUp.135.TGTTCT.I1	(n)PTB	I1(3')	new motifs
cisCons.YeoClustUp.136.CTTTT.I1	Motif.I1	I1(3')	new motifs
cisCons.YeoClustUp.137.ATTTGT.I1	Motif.I1	I1(3')	new motifs
cisCons.YeoClustUp.138.TGAGT.I1	Motif.I1	I1(3')	new motifs
cisCons.YeoClustUp.139.CCCCAG.I1	Motif.I1	I1(3')	new motifs
cisCons.YeoClustUp.140.TTGCAG.I1	Motif.I1	I1(3')	new motifs
cisCons.YeoClustUp.141.CTGAT.I1	Motif.I1	I1(3')	new motifs
cisCons.YeoClustUp.142.TAATA.I1	Motif.I1	I1(3')	new motifs
cisCons.YeoClustUp.143.TCTTA.I1	(n)PTB	I1(3')	new motifs
cisCons.YeoClustUp.144.ATTCT.I1	(n)PTB	I1(3')	new motifs
cisCons.YeoClustUp.145.ATCAAA.I1	Motif.I1	I1(3')	new motifs
cisCons.YeoClustUp.146.CTTTA.I1	Motif.I1	I1(3')	new motifs
cisCons.YeoClustUp.147.TTTAG.I1	Motif.I1	I1(3')	new motifs
cisCons.YeoClustUp.148.TTGCTG.I1	Motif.I1	I1(3')	new motifs
cisCons.YeoClustUp.149.TTTCAT.I1	Motif.I1	I1(3')	new motifs
cisCons.YeoClustUp.150.CTTTCA.I1	Motif.I1	I1(3')	new motifs
cisCons.YeoClustUp.151.TTCTC.I1	(n)PTB	I1(3')	new motifs
cisCons.YeoClustUp.152.TGCTT.I1	Motif.I1	I1(3')	new motifs
cisCons.YeoClustUp.153.TCTGC.I1	(n)PTB	I1(3')	new motifs
cisCons.YeoClustUp.154.CTGAA.I1	Motif.I1	I1(3')	new motifs
cisCons.YeoClustUp.155.GTAGGT.I1	Motif.I1	I1(3')	new motifs
cisCons.YeoClustUp.156.CTAAA.I1	Motif.I1	I1(3')	new motifs
cis.ESS.Burge.A	ESS	A	new motifs
cis.ESS.Burge.C1	ESS	C1	new motifs
cis.ESS.Burge.C2	ESS	C2	new motifs
cis.ESE.Burge.A	ESE	A	new motifs
cis.ESE.Burge.C1	ESE	C1	new motifs
cis.ESE.Burge.C2	ESE	C2	new motifs
cis.ESS.Chasin.A	ESS	A	new motifs
cis.ESS.Chasin.C1	ESS	C1	new motifs
cis.ESS.Chasin.C2	ESS	C2	new motifs
cis.ESE.Chasin.A	ESE	A	new motifs

cis.ESE.Chasin.C1	ESE	C1	new motifs
cis.ESE.Chasin.C2	ESE	C2	new motifs
cis.ShortSeq.AG.C2I2	NucFreq.C2I2	I2(3')	short motifs
cis.ShortSeq.AT.C2I2	NucFreq.C2I2	I2(3')	short motifs
cis.ShortSeq.CA.C2I2	NucFreq.C2I2	I2(3')	short motifs
cis.ShortSeq.CC.C2I2	NucFreq.C2I2	I2(3')	short motifs
cis.ShortSeq.CG.C2I2	NucFreq.C2I2	I2(3')	short motifs
cis.ShortSeq.CT.C2I2	NucFreq.C2I2	I2(3')	short motifs
cis.ShortSeq.GA.C2I2	NucFreq.C2I2	I2(3')	short motifs
cis.ShortSeq.GC.C2I2	NucFreq.C2I2	I2(3')	short motifs
cis.ShortSeq.GG.C2I2	NucFreq.C2I2	I2(3')	short motifs
cis.ShortSeq.GT.C2I2	NucFreq.C2I2	I2(3')	short motifs
cis.ShortSeq.TA.C2I2	NucFreq.C2I2	I2(3')	short motifs
cis.ShortSeq.TC.C2I2	NucFreq.C2I2	I2(3')	short motifs
cis.ShortSeq.TG.C2I2	NucFreq.C2I2	I2(3')	short motifs
cis.ShortSeq.TT.C2I2	NucFreq.C2I2	I2(3')	short motifs
cis.ShortSeq.A.C1	NucFreq.C1	C1	short motifs
cis.ShortSeq.C.C1	NucFreq.C1	C1	short motifs
cis.ShortSeq.G.C1	NucFreq.C1	C1	short motifs
cis.ShortSeq.T.C1	NucFreq.C1	C1	short motifs
cis.ShortSeq.AA.C1	NucFreq.C1	C1	short motifs
cis.ShortSeq.AC.C1	NucFreq.C1	C1	short motifs
cis.ShortSeq.AG.C1	NucFreq.C1	C1	short motifs
cis.ShortSeq.AT.C1	NucFreq.C1	C1	short motifs
cis.ShortSeq.CA.C1	NucFreq.C1	C1	short motifs
cis.ShortSeq.CC.C1	NucFreq.C1	C1	short motifs
cis.ShortSeq.CG.C1	NucFreq.C1	C1	short motifs
cis.ShortSeq.CT.C1	NucFreq.C1	C1	short motifs
cis.ShortSeq.GA.C1	NucFreq.C1	C1	short motifs
cis.ShortSeq.GC.C1	NucFreq.C1	C1	short motifs
cis.ShortSeq.GG.C1	NucFreq.C1	C1	short motifs
cis.ShortSeq.GT.C1	NucFreq.C1	C1	short motifs
cis.ShortSeq.TA.C1	NucFreq.C1	C1	short motifs
cis.ShortSeq.TC.C1	NucFreq.C1	C1	short motifs

cis.ShortSeq.TG.C1	NucFreq.C1	C1	short motifs
cis.ShortSeq.TT.C1	NucFreq.C1	C1	short motifs
cis.ShortSeq.AAA.C1	NucFreq.C1	C1	short motifs
cis.ShortSeq.AAC.C1	NucFreq.C1	C1	short motifs
cis.ShortSeq.AAG.C1	NucFreq.C1	C1	short motifs
cis.ShortSeq.AAT.C1	NucFreq.C1	C1	short motifs
cis.ShortSeq.ACA.C1	NucFreq.C1	C1	short motifs
cis.ShortSeq.ACC.C1	NucFreq.C1	C1	short motifs
cis.ShortSeq.ACG.C1	NucFreq.C1	C1	short motifs
cis.ShortSeq.ACT.C1	NucFreq.C1	C1	short motifs
cis.ShortSeq.AGA.C1	NucFreq.C1	C1	short motifs
cis.ShortSeq.AGC.C1	NucFreq.C1	C1	short motifs
cis.ShortSeq.AGG.C1	NucFreq.C1	C1	short motifs
cis.ShortSeq.AGT.C1	NucFreq.C1	C1	short motifs
cis.ShortSeq.ATA.C1	NucFreq.C1	C1	short motifs
cis.ShortSeq.ATC.C1	NucFreq.C1	C1	short motifs
cis.ShortSeq.ATG.C1	NucFreq.C1	C1	short motifs
cis.ShortSeq.ATT.C1	NucFreq.C1	C1	short motifs
cis.ShortSeq.CAA.C1	NucFreq.C1	C1	short motifs
cis.ShortSeq.CAC.C1	NucFreq.C1	C1	short motifs
cis.ShortSeq.CAG.C1	NucFreq.C1	C1	short motifs
cis.ShortSeq.CAT.C1	NucFreq.C1	C1	short motifs
cis.ShortSeq.CCA.C1	NucFreq.C1	C1	short motifs
cis.ShortSeq.CCC.C1	NucFreq.C1	C1	short motifs
cis.ShortSeq.CCG.C1	NucFreq.C1	C1	short motifs
cis.ShortSeq.CCT.C1	NucFreq.C1	C1	short motifs
cis.ShortSeq.CGA.C1	NucFreq.C1	C1	short motifs
cis.ShortSeq.CGC.C1	NucFreq.C1	C1	short motifs
cis.ShortSeq.CGG.C1	NucFreq.C1	C1	short motifs
cis.ShortSeq.CGT.C1	NucFreq.C1	C1	short motifs
cis.ShortSeq.CTA.C1	NucFreq.C1	C1	short motifs
cis.ShortSeq.CTC.C1	NucFreq.C1	C1	short motifs
cis.ShortSeq.CTG.C1	NucFreq.C1	C1	short motifs
cis.ShortSeq.CTT.C1	NucFreq.C1	C1	short motifs

cis.ShortSeq.GAA.C1	NucFreq.C1	C1	short motifs
cis.ShortSeq.GAC.C1	NucFreq.C1	C1	short motifs
cis.ShortSeq.GAG.C1	NucFreq.C1	C1	short motifs
cis.ShortSeq.GAT.C1	NucFreq.C1	C1	short motifs
cis.ShortSeq.GCA.C1	NucFreq.C1	C1	short motifs
cis.ShortSeq.GCC.C1	NucFreq.C1	C1	short motifs
cis.ShortSeq.GCG.C1	NucFreq.C1	C1	short motifs
cis.ShortSeq.GCT.C1	NucFreq.C1	C1	short motifs
cis.ShortSeq.GGA.C1	NucFreq.C1	C1	short motifs
cis.ShortSeq.GGC.C1	NucFreq.C1	C1	short motifs
cis.ShortSeq.GGG.C1	NucFreq.C1	C1	short motifs
cis.ShortSeq.GGT.C1	NucFreq.C1	C1	short motifs
cis.ShortSeq.GTA.C1	NucFreq.C1	C1	short motifs
cis.ShortSeq.GTC.C1	NucFreq.C1	C1	short motifs
cis.ShortSeq.GTG.C1	NucFreq.C1	C1	short motifs
cis.ShortSeq.GTT.C1	NucFreq.C1	C1	short motifs
cis.ShortSeq.TAA.C1	NucFreq.C1	C1	short motifs
cis.ShortSeq.TAC.C1	NucFreq.C1	C1	short motifs
cis.ShortSeq.TAG.C1	NucFreq.C1	C1	short motifs
cis.ShortSeq.TAT.C1	NucFreq.C1	C1	short motifs
cis.ShortSeq.TCA.C1	NucFreq.C1	C1	short motifs
cis.ShortSeq.TCC.C1	NucFreq.C1	C1	short motifs
cis.ShortSeq.TCG.C1	NucFreq.C1	C1	short motifs
cis.ShortSeq.TCT.C1	NucFreq.C1	C1	short motifs
cis.ShortSeq.TGA.C1	NucFreq.C1	C1	short motifs
cis.ShortSeq.TGC.C1	NucFreq.C1	C1	short motifs
cis.ShortSeq.TGG.C1	NucFreq.C1	C1	short motifs
cis.ShortSeq.TGT.C1	NucFreq.C1	C1	short motifs
cis.ShortSeq.TTA.C1	NucFreq.C1	C1	short motifs
cis.ShortSeq.TTC.C1	NucFreq.C1	C1	short motifs
cis.ShortSeq.TTG.C1	NucFreq.C1	C1	short motifs
cis.ShortSeq.TTT.C1	NucFreq.C1	C1	short motifs
cis.ShortSeq.A.C2	NucFreq.C2	C2	short motifs
cis.ShortSeq.C.C2	NucFreq.C2	C2	short motifs

cis.ShortSeq.G.C2	NucFreq.C2	C2	short motifs
cis.ShortSeq.T.C2	NucFreq.C2	C2	short motifs
cis.ShortSeq.AA.C2	NucFreq.C2	C2	short motifs
cis.ShortSeq.AC.C2	NucFreq.C2	C2	short motifs
cis.ShortSeq.AG.C2	NucFreq.C2	C2	short motifs
cis.ShortSeq.AT.C2	NucFreq.C2	C2	short motifs
cis.ShortSeq.CA.C2	NucFreq.C2	C2	short motifs
cis.ShortSeq.CC.C2	NucFreq.C2	C2	short motifs
cis.ShortSeq.CG.C2	NucFreq.C2	C2	short motifs
cis.ShortSeq.CT.C2	NucFreq.C2	C2	short motifs
cis.ShortSeq.GA.C2	NucFreq.C2	C2	short motifs
cis.ShortSeq.GC.C2	NucFreq.C2	C2	short motifs
cis.ShortSeq.GG.C2	NucFreq.C2	C2	short motifs
cis.ShortSeq.GT.C2	NucFreq.C2	C2	short motifs
cis.ShortSeq.TA.C2	NucFreq.C2	C2	short motifs
cis.ShortSeq.TC.C2	NucFreq.C2	C2	short motifs
cis.ShortSeq.TG.C2	NucFreq.C2	C2	short motifs
cis.ShortSeq.TT.C2	NucFreq.C2	C2	short motifs
cis.ShortSeq.AAA.C2	NucFreq.C2	C2	short motifs
cis.ShortSeq.AAC.C2	NucFreq.C2	C2	short motifs
cis.ShortSeq.AAG.C2	NucFreq.C2	C2	short motifs
cis.ShortSeq.AAT.C2	NucFreq.C2	C2	short motifs
cis.ShortSeq.ACA.C2	NucFreq.C2	C2	short motifs
cis.ShortSeq.ACC.C2	NucFreq.C2	C2	short motifs
cis.ShortSeq.ACG.C2	NucFreq.C2	C2	short motifs
cis.ShortSeq.ACT.C2	NucFreq.C2	C2	short motifs
cis.ShortSeq.AGA.C2	NucFreq.C2	C2	short motifs
cis.ShortSeq.AGC.C2	NucFreq.C2	C2	short motifs
cis.ShortSeq.AGG.C2	NucFreq.C2	C2	short motifs
cis.ShortSeq.AGT.C2	NucFreq.C2	C2	short motifs
cis.ShortSeq.ATA.C2	NucFreq.C2	C2	short motifs
cis.ShortSeq.ATC.C2	NucFreq.C2	C2	short motifs
cis.ShortSeq.ATG.C2	NucFreq.C2	C2	short motifs
cis.ShortSeq.ATT.C2	NucFreq.C2	C2	short motifs

cis.ShortSeq.CAA.C2	NucFreq.C2	C2	short motifs
cis.ShortSeq.CAC.C2	NucFreq.C2	C2	short motifs
cis.ShortSeq.CAG.C2	NucFreq.C2	C2	short motifs
cis.ShortSeq.CAT.C2	NucFreq.C2	C2	short motifs
cis.ShortSeq.CCA.C2	NucFreq.C2	C2	short motifs
cis.ShortSeq.CCC.C2	NucFreq.C2	C2	short motifs
cis.ShortSeq.CCG.C2	NucFreq.C2	C2	short motifs
cis.ShortSeq.CCT.C2	NucFreq.C2	C2	short motifs
cis.ShortSeq.CGA.C2	NucFreq.C2	C2	short motifs
cis.ShortSeq.CGC.C2	NucFreq.C2	C2	short motifs
cis.ShortSeq.CGG.C2	NucFreq.C2	C2	short motifs
cis.ShortSeq.CGT.C2	NucFreq.C2	C2	short motifs
cis.ShortSeq.CTA.C2	NucFreq.C2	C2	short motifs
cis.ShortSeq.CTC.C2	NucFreq.C2	C2	short motifs
cis.ShortSeq.CTG.C2	NucFreq.C2	C2	short motifs
cis.ShortSeq.CTT.C2	NucFreq.C2	C2	short motifs
cis.ShortSeq.GAA.C2	NucFreq.C2	C2	short motifs
cis.ShortSeq.GAC.C2	NucFreq.C2	C2	short motifs
cis.ShortSeq.GAG.C2	NucFreq.C2	C2	short motifs
cis.ShortSeq.GAT.C2	NucFreq.C2	C2	short motifs
cis.ShortSeq.GCA.C2	NucFreq.C2	C2	short motifs
cis.ShortSeq.GCC.C2	NucFreq.C2	C2	short motifs
cis.ShortSeq.GCG.C2	NucFreq.C2	C2	short motifs
cis.ShortSeq.GCT.C2	NucFreq.C2	C2	short motifs
cis.ShortSeq.GGA.C2	NucFreq.C2	C2	short motifs
cis.ShortSeq.GGC.C2	NucFreq.C2	C2	short motifs
cis.ShortSeq.GGG.C2	NucFreq.C2	C2	short motifs
cis.ShortSeq.GGT.C2	NucFreq.C2	C2	short motifs
cis.ShortSeq.GTA.C2	NucFreq.C2	C2	short motifs
cis.ShortSeq.GTC.C2	NucFreq.C2	C2	short motifs
cis.ShortSeq.GTG.C2	NucFreq.C2	C2	short motifs
cis.ShortSeq.GTT.C2	NucFreq.C2	C2	short motifs
cis.ShortSeq.TAA.C2	NucFreq.C2	C2	short motifs
cis.ShortSeq.TAC.C2	NucFreq.C2	C2	short motifs

cis.ShortSeq.TAG.C2	NucFreq.C2	C2	short motifs
cis.ShortSeq.TAT.C2	NucFreq.C2	C2	short motifs
cis.ShortSeq.TCA.C2	NucFreq.C2	C2	short motifs
cis.ShortSeq.TCC.C2	NucFreq.C2	C2	short motifs
cis.ShortSeq.TCG.C2	NucFreq.C2	C2	short motifs
cis.ShortSeq.TCT.C2	NucFreq.C2	C2	short motifs
cis.ShortSeq.TGA.C2	NucFreq.C2	C2	short motifs
cis.ShortSeq.TGC.C2	NucFreq.C2	C2	short motifs
cis.ShortSeq.TGG.C2	NucFreq.C2	C2	short motifs
cis.ShortSeq.TGT.C2	NucFreq.C2	C2	short motifs
cis.ShortSeq.TTA.C2	NucFreq.C2	C2	short motifs
cis.ShortSeq.TTC.C2	NucFreq.C2	C2	short motifs
cis.ShortSeq.TTG.C2	NucFreq.C2	C2	short motifs
cis.ShortSeq.TTT.C2	NucFreq.C2	C2	short motifs
cis.ShortSeq.A.A	NucFreq.A	A	short motifs
cis.ShortSeq.C.A	NucFreq.A	A	short motifs
cis.ShortSeq.G.A	NucFreq.A	A	short motifs
cis.ShortSeq.T.A	NucFreq.A	A	short motifs
cis.ShortSeq.AA.A	NucFreq.A	A	short motifs
cis.ShortSeq.AC.A	NucFreq.A	A	short motifs
cis.ShortSeq.AG.A	NucFreq.A	A	short motifs
cis.ShortSeq.AT.A	NucFreq.A	A	short motifs
cis.ShortSeq.CA.A	NucFreq.A	A	short motifs
cis.ShortSeq.CC.A	NucFreq.A	A	short motifs
cis.ShortSeq.CG.A	NucFreq.A	A	short motifs
cis.ShortSeq.CT.A	NucFreq.A	A	short motifs
cis.ShortSeq.GA.A	NucFreq.A	A	short motifs
cis.ShortSeq.GC.A	NucFreq.A	A	short motifs
cis.ShortSeq.GG.A	NucFreq.A	A	short motifs
cis.ShortSeq.GT.A	NucFreq.A	A	short motifs
cis.ShortSeq.TA.A	NucFreq.A	A	short motifs
cis.ShortSeq.TC.A	NucFreq.A	A	short motifs
cis.ShortSeq.TG.A	NucFreq.A	A	short motifs
cis.ShortSeq.TT.A	NucFreq.A	A	short motifs

cis.ShortSeq.AAA.A	NucFreq.A	A	short motifs
cis.ShortSeq.AAC.A	NucFreq.A	A	short motifs
cis.ShortSeq.AAG.A	NucFreq.A	A	short motifs
cis.ShortSeq.AAT.A	NucFreq.A	A	short motifs
cis.ShortSeq.ACA.A	NucFreq.A	A	short motifs
cis.ShortSeq.ACC.A	NucFreq.A	A	short motifs
cis.ShortSeq.ACG.A	NucFreq.A	A	short motifs
cis.ShortSeq.ACT.A	NucFreq.A	A	short motifs
cis.ShortSeq.AGA.A	NucFreq.A	A	short motifs
cis.ShortSeq.AGC.A	NucFreq.A	A	short motifs
cis.ShortSeq.AGG.A	NucFreq.A	A	short motifs
cis.ShortSeq.AGT.A	NucFreq.A	A	short motifs
cis.ShortSeq.ATA.A	NucFreq.A	A	short motifs
cis.ShortSeq.ATC.A	NucFreq.A	A	short motifs
cis.ShortSeq.ATG.A	NucFreq.A	A	short motifs
cis.ShortSeq.ATT.A	NucFreq.A	A	short motifs
cis.ShortSeq.CAA.A	NucFreq.A	A	short motifs
cis.ShortSeq.CAC.A	NucFreq.A	A	short motifs
cis.ShortSeq.CAG.A	NucFreq.A	A	short motifs
cis.ShortSeq.CAT.A	NucFreq.A	A	short motifs
cis.ShortSeq.CCA.A	NucFreq.A	A	short motifs
cis.ShortSeq.CCC.A	NucFreq.A	A	short motifs
cis.ShortSeq.CCG.A	NucFreq.A	A	short motifs
cis.ShortSeq.CCT.A	NucFreq.A	A	short motifs
cis.ShortSeq.CGA.A	NucFreq.A	A	short motifs
cis.ShortSeq.CGC.A	NucFreq.A	A	short motifs
cis.ShortSeq.CGG.A	NucFreq.A	A	short motifs
cis.ShortSeq.CGT.A	NucFreq.A	A	short motifs
cis.ShortSeq.CTA.A	NucFreq.A	A	short motifs
cis.ShortSeq.CTC.A	NucFreq.A	A	short motifs
cis.ShortSeq.CTG.A	NucFreq.A	A	short motifs
cis.ShortSeq.CTT.A	NucFreq.A	A	short motifs
cis.ShortSeq.GAA.A	NucFreq.A	A	short motifs
cis.ShortSeq.GAC.A	NucFreq.A	A	short motifs

cis.ShortSeq.GAG.A	NucFreq.A	A	short motifs
cis.ShortSeq.GAT.A	NucFreq.A	A	short motifs
cis.ShortSeq.GCA.A	NucFreq.A	A	short motifs
cis.ShortSeq.GCC.A	NucFreq.A	A	short motifs
cis.ShortSeq.GCG.A	NucFreq.A	A	short motifs
cis.ShortSeq.GCT.A	NucFreq.A	A	short motifs
cis.ShortSeq.GGA.A	NucFreq.A	A	short motifs
cis.ShortSeq.GGC.A	NucFreq.A	A	short motifs
cis.ShortSeq.GGG.A	NucFreq.A	A	short motifs
cis.ShortSeq.GGT.A	NucFreq.A	A	short motifs
cis.ShortSeq.GTA.A	NucFreq.A	A	short motifs
cis.ShortSeq.GTC.A	NucFreq.A	A	short motifs
cis.ShortSeq.GTG.A	NucFreq.A	A	short motifs
cis.ShortSeq.GTT.A	NucFreq.A	A	short motifs
cis.ShortSeq.TAA.A	NucFreq.A	A	short motifs
cis.ShortSeq.TAC.A	NucFreq.A	A	short motifs
cis.ShortSeq.TAG.A	NucFreq.A	A	short motifs
cis.ShortSeq.TAT.A	NucFreq.A	A	short motifs
cis.ShortSeq.TCA.A	NucFreq.A	A	short motifs
cis.ShortSeq.TCC.A	NucFreq.A	A	short motifs
cis.ShortSeq.TCG.A	NucFreq.A	A	short motifs
cis.ShortSeq.TCT.A	NucFreq.A	A	short motifs
cis.ShortSeq.TGA.A	NucFreq.A	A	short motifs
cis.ShortSeq.TGC.A	NucFreq.A	A	short motifs
cis.ShortSeq.TGG.A	NucFreq.A	A	short motifs
cis.ShortSeq.TGT.A	NucFreq.A	A	short motifs
cis.ShortSeq.TTA.A	NucFreq.A	A	short motifs
cis.ShortSeq.TTC.A	NucFreq.A	A	short motifs
cis.ShortSeq.TTG.A	NucFreq.A	A	short motifs
cis.ShortSeq.TTT.A	NucFreq.A	A	short motifs
cis.ShortSeq.A.I2	NucFreq.I2	I2(5')	short motifs
cis.ShortSeq.C.I2	NucFreq.I2	I2(5')	short motifs
cis.ShortSeq.G.I2	NucFreq.I2	I2(5')	short motifs
cis.ShortSeq.T.I2	NucFreq.I2	I2(5')	short motifs

cis.ShortSeq.AA.I2	NucFreq.I2	I2(5')	short motifs
cis.ShortSeq.AC.I2	NucFreq.I2	I2(5')	short motifs
cis.ShortSeq.AG.I2	NucFreq.I2	I2(5')	short motifs
cis.ShortSeq.AT.I2	NucFreq.I2	I2(5')	short motifs
cis.ShortSeq.CA.I2	NucFreq.I2	I2(5')	short motifs
cis.ShortSeq.CC.I2	NucFreq.I2	I2(5')	short motifs
cis.ShortSeq.CG.I2	NucFreq.I2	I2(5')	short motifs
cis.ShortSeq.CT.I2	NucFreq.I2	I2(5')	short motifs
cis.ShortSeq.GA.I2	NucFreq.I2	I2(5')	short motifs
cis.ShortSeq.GC.I2	NucFreq.I2	I2(5')	short motifs
cis.ShortSeq.GG.I2	NucFreq.I2	I2(5')	short motifs
cis.ShortSeq.GT.I2	NucFreq.I2	I2(5')	short motifs
cis.ShortSeq.TA.I2	NucFreq.I2	I2(5')	short motifs
cis.ShortSeq.TC.I2	NucFreq.I2	I2(5')	short motifs
cis.ShortSeq.TG.I2	NucFreq.I2	I2(5')	short motifs
cis.ShortSeq.TT.I2	NucFreq.I2	I2(5')	short motifs
cis.ShortSeq.AAA.I2	NucFreq.I2	I2(5')	short motifs
cis.ShortSeq.AAC.I2	NucFreq.I2	I2(5')	short motifs
cis.ShortSeq.AAG.I2	NucFreq.I2	I2(5')	short motifs
cis.ShortSeq.AAT.I2	NucFreq.I2	I2(5')	short motifs
cis.ShortSeq.ACA.I2	NucFreq.I2	I2(5')	short motifs
cis.ShortSeq.ACC.I2	NucFreq.I2	I2(5')	short motifs
cis.ShortSeq.ACG.I2	NucFreq.I2	I2(5')	short motifs
cis.ShortSeq.ACT.I2	NucFreq.I2	I2(5')	short motifs
cis.ShortSeq.AGA.I2	NucFreq.I2	I2(5')	short motifs
cis.ShortSeq.AGC.I2	NucFreq.I2	I2(5')	short motifs
cis.ShortSeq.AGG.I2	NucFreq.I2	I2(5')	short motifs
cis.ShortSeq.AGT.I2	NucFreq.I2	I2(5')	short motifs
cis.ShortSeq.ATA.I2	NucFreq.I2	I2(5')	short motifs
cis.ShortSeq.ATC.I2	NucFreq.I2	I2(5')	short motifs
cis.ShortSeq.ATG.I2	NucFreq.I2	I2(5')	short motifs
cis.ShortSeq.ATT.I2	NucFreq.I2	I2(5')	short motifs
cis.ShortSeq.CAA.I2	NucFreq.I2	I2(5')	short motifs
cis.ShortSeq.CAC.I2	NucFreq.I2	I2(5')	short motifs

cis.ShortSeq.CAG.I2	NucFreq.I2	I2(5')	short motifs
cis.ShortSeq.CAT.I2	NucFreq.I2	I2(5')	short motifs
cis.ShortSeq.CCA.I2	NucFreq.I2	I2(5')	short motifs
cis.ShortSeq.CCC.I2	NucFreq.I2	I2(5')	short motifs
cis.ShortSeq.CCG.I2	NucFreq.I2	I2(5')	short motifs
cis.ShortSeq.CCT.I2	NucFreq.I2	I2(5')	short motifs
cis.ShortSeq.CGA.I2	NucFreq.I2	I2(5')	short motifs
cis.ShortSeq.CGC.I2	NucFreq.I2	I2(5')	short motifs
cis.ShortSeq.CGG.I2	NucFreq.I2	I2(5')	short motifs
cis.ShortSeq.CGT.I2	NucFreq.I2	I2(5')	short motifs
cis.ShortSeq.CTA.I2	NucFreq.I2	I2(5')	short motifs
cis.ShortSeq.CTC.I2	NucFreq.I2	I2(5')	short motifs
cis.ShortSeq.CTG.I2	NucFreq.I2	I2(5')	short motifs
cis.ShortSeq.CTT.I2	NucFreq.I2	I2(5')	short motifs
cis.ShortSeq.GAA.I2	NucFreq.I2	I2(5')	short motifs
cis.ShortSeq.GAC.I2	NucFreq.I2	I2(5')	short motifs
cis.ShortSeq.GAG.I2	NucFreq.I2	I2(5')	short motifs
cis.ShortSeq.GAT.I2	NucFreq.I2	I2(5')	short motifs
cis.ShortSeq.GCA.I2	NucFreq.I2	I2(5')	short motifs
cis.ShortSeq.GCC.I2	NucFreq.I2	I2(5')	short motifs
cis.ShortSeq.GCG.I2	NucFreq.I2	I2(5')	short motifs
cis.ShortSeq.GCT.I2	NucFreq.I2	I2(5')	short motifs
cis.ShortSeq.GGA.I2	NucFreq.I2	I2(5')	short motifs
cis.ShortSeq.GGC.I2	NucFreq.I2	I2(5')	short motifs
cis.ShortSeq.GGG.I2	NucFreq.I2	I2(5')	short motifs
cis.ShortSeq.GGT.I2	NucFreq.I2	I2(5')	short motifs
cis.ShortSeq.GTA.I2	NucFreq.I2	I2(5')	short motifs
cis.ShortSeq.GTC.I2	NucFreq.I2	I2(5')	short motifs
cis.ShortSeq.GTG.I2	NucFreq.I2	I2(5')	short motifs
cis.ShortSeq.GTT.I2	NucFreq.I2	I2(5')	short motifs
cis.ShortSeq.TAA.I2	NucFreq.I2	I2(5')	short motifs
cis.ShortSeq.TAC.I2	NucFreq.I2	I2(5')	short motifs
cis.ShortSeq.TAG.I2	NucFreq.I2	I2(5')	short motifs
cis.ShortSeq.TAT.I2	NucFreq.I2	I2(5')	short motifs

cis.ShortSeq.TCA.I2	NucFreq.I2	I2(5')	short motifs
cis.ShortSeq.TCC.I2	NucFreq.I2	I2(5')	short motifs
cis.ShortSeq.TCG.I2	NucFreq.I2	I2(5')	short motifs
cis.ShortSeq.TCT.I2	NucFreq.I2	I2(5')	short motifs
cis.ShortSeq.TGA.I2	NucFreq.I2	I2(5')	short motifs
cis.ShortSeq.TGC.I2	NucFreq.I2	I2(5')	short motifs
cis.ShortSeq.TGG.I2	NucFreq.I2	I2(5')	short motifs
cis.ShortSeq.TGT.I2	NucFreq.I2	I2(5')	short motifs
cis.ShortSeq.TTA.I2	NucFreq.I2	I2(5')	short motifs
cis.ShortSeq.TTC.I2	NucFreq.I2	I2(5')	short motifs
cis.ShortSeq.TTG.I2	NucFreq.I2	I2(5')	short motifs
cis.ShortSeq.TTT.I2	NucFreq.I2	I2(5')	short motifs
cis.ShortSeq.A.I1	NucFreq.I1	I1(3')	short motifs
cis.ShortSeq.C.I1	NucFreq.I1	I1(3')	short motifs
cis.ShortSeq.G.I1	NucFreq.I1	I1(3')	short motifs
cis.ShortSeq.T.I1	NucFreq.I1	I1(3')	short motifs
cis.ShortSeq.AA.I1	NucFreq.I1	I1(3')	short motifs
cis.ShortSeq.AC.I1	NucFreq.I1	I1(3')	short motifs
cis.ShortSeq.AG.I1	NucFreq.I1	I1(3')	short motifs
cis.ShortSeq.AT.I1	NucFreq.I1	I1(3')	short motifs
cis.ShortSeq.CA.I1	NucFreq.I1	I1(3')	short motifs
cis.ShortSeq.CC.I1	NucFreq.I1	I1(3')	short motifs
cis.ShortSeq.CG.I1	NucFreq.I1	I1(3')	short motifs
cis.ShortSeq.CT.I1	NucFreq.I1	I1(3')	short motifs
cis.ShortSeq.GA.I1	NucFreq.I1	I1(3')	short motifs
cis.ShortSeq.GC.I1	NucFreq.I1	I1(3')	short motifs
cis.ShortSeq.GG.I1	NucFreq.I1	I1(3')	short motifs
cis.ShortSeq.GT.I1	NucFreq.I1	I1(3')	short motifs
cis.ShortSeq.TA.I1	NucFreq.I1	I1(3')	short motifs
cis.ShortSeq.TC.I1	NucFreq.I1	I1(3')	short motifs
cis.ShortSeq.TG.I1	NucFreq.I1	I1(3')	short motifs
cis.ShortSeq.TT.I1	NucFreq.I1	I1(3')	short motifs
cis.ShortSeq.AAA.I1	NucFreq.I1	I1(3')	short motifs
cis.ShortSeq.AAC.I1	NucFreq.I1	I1(3')	short motifs

cis.ShortSeq.AAG.I1	NucFreq.I1	I1(3')	short motifs
cis.ShortSeq.AAT.I1	NucFreq.I1	I1(3')	short motifs
cis.ShortSeq.ACA.I1	NucFreq.I1	I1(3')	short motifs
cis.ShortSeq.ACC.I1	NucFreq.I1	I1(3')	short motifs
cis.ShortSeq.ACG.I1	NucFreq.I1	I1(3')	short motifs
cis.ShortSeq.ACT.I1	NucFreq.I1	I1(3')	short motifs
cis.ShortSeq.AGA.I1	NucFreq.I1	I1(3')	short motifs
cis.ShortSeq.AGC.I1	NucFreq.I1	I1(3')	short motifs
cis.ShortSeq.AGG.I1	NucFreq.I1	I1(3')	short motifs
cis.ShortSeq.AGT.I1	NucFreq.I1	I1(3')	short motifs
cis.ShortSeq.ATA.I1	NucFreq.I1	I1(3')	short motifs
cis.ShortSeq.ATC.I1	NucFreq.I1	I1(3')	short motifs
cis.ShortSeq.ATG.I1	NucFreq.I1	I1(3')	short motifs
cis.ShortSeq.ATT.I1	NucFreq.I1	I1(3')	short motifs
cis.ShortSeq.CAA.I1	NucFreq.I1	I1(3')	short motifs
cis.ShortSeq.CAC.I1	NucFreq.I1	I1(3')	short motifs
cis.ShortSeq.CAG.I1	NucFreq.I1	I1(3')	short motifs
cis.ShortSeq.CAT.I1	NucFreq.I1	I1(3')	short motifs
cis.ShortSeq.CCA.I1	NucFreq.I1	I1(3')	short motifs
cis.ShortSeq.CCC.I1	NucFreq.I1	I1(3')	short motifs
cis.ShortSeq.CCG.I1	NucFreq.I1	I1(3')	short motifs
cis.ShortSeq.CCT.I1	NucFreq.I1	I1(3')	short motifs
cis.ShortSeq.CGA.I1	NucFreq.I1	I1(3')	short motifs
cis.ShortSeq.CGC.I1	NucFreq.I1	I1(3')	short motifs
cis.ShortSeq.CGG.I1	NucFreq.I1	I1(3')	short motifs
cis.ShortSeq.CGT.I1	NucFreq.I1	I1(3')	short motifs
cis.ShortSeq.CTA.I1	NucFreq.I1	I1(3')	short motifs
cis.ShortSeq.CTC.I1	NucFreq.I1	I1(3')	short motifs
cis.ShortSeq.CTG.I1	NucFreq.I1	I1(3')	short motifs
cis.ShortSeq.CTT.I1	NucFreq.I1	I1(3')	short motifs
cis.ShortSeq.GAA.I1	NucFreq.I1	I1(3')	short motifs
cis.ShortSeq.GAC.I1	NucFreq.I1	I1(3')	short motifs
cis.ShortSeq.GAG.I1	NucFreq.I1	I1(3')	short motifs
cis.ShortSeq.GAT.I1	NucFreq.I1	I1(3')	short motifs

cis.ShortSeq.GCA.I1	NucFreq.I1	I1(3')	short motifs
cis.ShortSeq.GCC.I1	NucFreq.I1	I1(3')	short motifs
cis.ShortSeq.GCG.I1	NucFreq.I1	I1(3')	short motifs
cis.ShortSeq.GCT.I1	NucFreq.I1	I1(3')	short motifs
cis.ShortSeq.GGA.I1	NucFreq.I1	I1(3')	short motifs
cis.ShortSeq.GGC.I1	NucFreq.I1	I1(3')	short motifs
cis.ShortSeq.GGG.I1	NucFreq.I1	I1(3')	short motifs
cis.ShortSeq.GGT.I1	NucFreq.I1	I1(3')	short motifs
cis.ShortSeq.GTA.I1	NucFreq.I1	I1(3')	short motifs
cis.ShortSeq.GTC.I1	NucFreq.I1	I1(3')	short motifs
cis.ShortSeq.GTG.I1	NucFreq.I1	I1(3')	short motifs
cis.ShortSeq.GTT.I1	NucFreq.I1	I1(3')	short motifs
cis.ShortSeq.TAA.I1	NucFreq.I1	I1(3')	short motifs
cis.ShortSeq.TAC.I1	NucFreq.I1	I1(3')	short motifs
cis.ShortSeq.TAG.I1	NucFreq.I1	I1(3')	short motifs
cis.ShortSeq.TAT.I1	NucFreq.I1	I1(3')	short motifs
cis.ShortSeq.TCA.I1	NucFreq.I1	I1(3')	short motifs
cis.ShortSeq.TCC.I1	NucFreq.I1	I1(3')	short motifs
cis.ShortSeq.TCG.I1	NucFreq.I1	I1(3')	short motifs
cis.ShortSeq.TCT.I1	NucFreq.I1	I1(3')	short motifs
cis.ShortSeq.TGA.I1	NucFreq.I1	I1(3')	short motifs
cis.ShortSeq.TGC.I1	NucFreq.I1	I1(3')	short motifs
cis.ShortSeq.TGG.I1	NucFreq.I1	I1(3')	short motifs
cis.ShortSeq.TGT.I1	NucFreq.I1	I1(3')	short motifs
cis.ShortSeq.TTA.I1	NucFreq.I1	I1(3')	short motifs
cis.ShortSeq.TTC.I1	NucFreq.I1	I1(3')	short motifs
cis.ShortSeq.TTG.I1	NucFreq.I1	I1(3')	short motifs
cis.ShortSeq.TTT.I1	NucFreq.I1	I1(3')	short motifs
cis.ShortSeq.A.C1I1	NucFreq.C1I1	I1(5')	short motifs
cis.ShortSeq.C.C1I1	NucFreq.C1I1	I1(5')	short motifs
cis.ShortSeq.G.C1I1	NucFreq.C1I1	I1(5')	short motifs
cis.ShortSeq.T.C1I1	NucFreq.C1I1	I1(5')	short motifs
cis.ShortSeq.AA.C1I1	NucFreq.C1I1	I1(5')	short motifs
cis.ShortSeq.AC.C1I1	NucFreq.C1I1	I1(5')	short motifs

cis.ShortSeq.AG.C1I1	NucFreq.C1I1	I1(5')	short motifs
cis.ShortSeq.AT.C1I1	NucFreq.C1I1	I1(5')	short motifs
cis.ShortSeq.CA.C1I1	NucFreq.C1I1	I1(5')	short motifs
cis.ShortSeq.CC.C1I1	NucFreq.C1I1	I1(5')	short motifs
cis.ShortSeq.CG.C1I1	NucFreq.C1I1	I1(5')	short motifs
cis.ShortSeq.CT.C1I1	NucFreq.C1I1	I1(5')	short motifs
cis.ShortSeq.GA.C1I1	NucFreq.C1I1	I1(5')	short motifs
cis.ShortSeq.GC.C1I1	NucFreq.C1I1	I1(5')	short motifs
cis.ShortSeq.GG.C1I1	NucFreq.C1I1	I1(5')	short motifs
cis.ShortSeq.GT.C1I1	NucFreq.C1I1	I1(5')	short motifs
cis.ShortSeq.TA.C1I1	NucFreq.C1I1	I1(5')	short motifs
cis.ShortSeq.TC.C1I1	NucFreq.C1I1	I1(5')	short motifs
cis.ShortSeq.TG.C1I1	NucFreq.C1I1	I1(5')	short motifs
cis.ShortSeq.TT.C1I1	NucFreq.C1I1	I1(5')	short motifs
cis.ShortSeq.A.C2I2	NucFreq.C2I2	I2(3')	short motifs
cis.ShortSeq.C.C2I2	NucFreq.C2I2	I2(3')	short motifs
cis.ShortSeq.G.C2I2	NucFreq.C2I2	I2(3')	short motifs
cis.ShortSeq.T.C2I2	NucFreq.C2I2	I2(3')	short motifs
cis.ShortSeq.AA.C2I2	NucFreq.C2I2	I2(3')	short motifs
JuncScore.C1I1	JuncScore	NaN	transcript structure features
JuncScore.C2I2	JuncScore	NaN	transcript structure features
Len.C1	Len	C1	transcript structure features
Len.A	Len	A	transcript structure features
Len.C2	Len	C2	transcript structure features
Len.I1	Len	I1(3')	transcript structure features
Len.I2	Len	I2(5')	transcript structure features
Len.Ratio.A_I1	Len	NaN	transcript structure features
Len.Ratio.A_I2	Len	NaN	transcript structure features
Len.Ratio.I1_I2	Len	NaN	transcript structure features
Frame.shift.NaN	Shift	NaN	transcript structure features
AltAGpos	AltAGpos	I1(3')	transcript structure features
AltGTpos	AltGTpos	I2(5')	transcript structure features
Cons.MeanP1_100.C1I1	Cons	I1(5')	transcript structure features
Cons.MeanP1_100.C2I2	Cons	I2(3')	transcript structure features

Cons.MeanP1_100.I1	Cons	I1(3')	transcript structure features
Cons.MeanP1_100.I2	Cons	I2(5')	transcript structure features
SecStr.maxJunc.C1I1	SecStr.C1I1	I1(5')	transcript structure features
SecStr.avgJunc.C1I1	SecStr.C1I1	I1(5')	transcript structure features
SecStr.maxJunc.AI1	SecStr.I1	I1(3')	transcript structure features
SecStr.avgJunc.AI1	SecStr.I1	I1(3')	transcript structure features
Frame.noShift.NaN	Shift	NaN	transcript structure features
SecStr.maxJunc.AI2	SecStr.I2	I2(5')	transcript structure features
SecStr.avgJunc.AI2	SecStr.I2	I2(5')	transcript structure features
SecStr.maxJunc.C2I2	SecStr.C2I2	I2(3')	transcript structure features
SecStr.avgJunc.C2I2	SecStr.C2I2	I2(3')	transcript structure features
SecStr.max1_70.C1I1	SecStr.C1I1	I1(5')	transcript structure features
SecStr.avg1_70.C1I1	SecStr.C1I1	I1(5')	transcript structure features
SecStr.max71_140.C1I1	SecStr.C1I1	I1(5')	transcript structure features
SecStr.avg71_140.C1I1	SecStr.C1I1	I1(5')	transcript structure features
SecStr.max141_210.C1I1	SecStr.C1I1	I1(5')	transcript structure features
SecStr.avg141_210.C1I1	SecStr.C1I1	I1(5')	transcript structure features
PTC.NotMined.NaN	PTC	NaN	transcript structure features
SecStr.max1_70.AI2	SecStr.I2	I2(5')	transcript structure features
SecStr.avg1_70.AI2	SecStr.I2	I2(5')	transcript structure features
SecStr.max71_140.AI2	SecStr.I2	I2(5')	transcript structure features
SecStr.avg71_140.AI2	SecStr.I2	I2(5')	transcript structure features
SecStr.max141_210.AI2	SecStr.I2	I2(5')	transcript structure features
SecStr.avg141_210.AI2	SecStr.I2	I2(5')	transcript structure features
SecStr.max1_70.AI1	SecStr.I1	I1(3')	transcript structure features
SecStr.avg1_70.AI1	SecStr.I1	I1(3')	transcript structure features
SecStr.max71_140.AI1	SecStr.I1	I1(3')	transcript structure features
SecStr.avg71_140.AI1	SecStr.I1	I1(3')	transcript structure features
PTC.ExcludedFromAnalysis.NaN	PTC	NaN	transcript structure features
SecStr.max141_210.AI1	SecStr.I1	I1(3')	transcript structure features
SecStr.avg141_210.AI1	SecStr.I1	I1(3')	transcript structure features
SecStr.max1_70.C2I2	SecStr.C2I2	I2(3')	transcript structure features
SecStr.avg1_70.C2I2	SecStr.C2I2	I2(3')	transcript structure features
SecStr.max71_140.C2I2	SecStr.C2I2	I2(3')	transcript structure features

SecStr.avg71_140.C2I2	SecStr.C2I2	I2(3')	transcript structure features
SecStr.max141_210.C2I2	SecStr.C2I2	I2(3')	transcript structure features
SecStr.avg141_210.C2I2	SecStr.C2I2	I2(3')	transcript structure features
PTC.NoPTC.NaN	PTC	NaN	transcript structure features
PTC.UponInclusion.NaN	PTC	NaN	transcript structure features
PTC.UponeExclusion.NaN	PTC	NaN	transcript structure features
JuncScore.AI1	JuncScore	NaN	transcript structure features
JuncScore.AI2	JuncScore	NaN	transcript structure features

Table S3 - The identified key features and corresponding MCCs on each data set

	CNS		Muscle		Embryo		Digestive	
	MCC	# Features	MCC	# Features	MCC	# Features	MCC	# Features
Dataset1	0.353	44	0.298	59	0.237	56	0.288	134
Dataset2	0.377	42	0.375	129	0.254	183	0.272	127
Dataset3	0.407	18	0.345	50	0.239	81	0.218	112
Dataset4	0.371	25	0.282	187	0.227	37	0.296	176
Dataset5	0.354	37	0.338	78	0.272	113	0.308	156
Dataset6	0.377	39	0.330	183	0.249	53	0.226	76
MCC (Mean ±SD*)	0.373 ±0.019	-	0.328 ±0.034	-	0.246 ±0.016	-	0.268 ±0.038	-
# Combined Features	-	122	-	391	-	343	-	464

*SD: standard deviation

Table S4 - The key features for alternative splicing in CNS, muscle, Embryo and digestive tissues

**Key features in CNS tissue**	**Key features in muscle tissue**	**Key features in Embryo tissue**	**Key features in digestive tissue**
Frame.shift.NaN	Frame.shift.NaN	cis.Fox.v2.C2I2	cis.Fox.v1.C2I2
cis.MBNL.v3.I2	JuncScore.C1I1	cis.Fox.v3.C2I2	cis.Fox.v2.I2
cisCons.Nova.AI2_C2I2	cis.Fox.v2.I2	cis.Nova.NISS2	cis.Fox.v3.I2
cisCons.Fox.v1.I1I2	cis.GUrich.v1.I2	cis.Fox.v2.I2	cis.GUrich.v2.I2
cisCons.PTB.v2.I1I2	cis.GUrich.v2.I2	cis.GUrich.v2.I2	cis.Tra2.I2
cisCons.PTB.v3.I1I2	cis.GUrich.v4.I2	cis.GUrich.v3.I2	cis.UGrich.v1.I2
cisCons.Fox.v3.C1I1	cis.Tra2.I2	cis.UGrich.v2.I2	cis.CUGBP.n2.I2
cisCons.Fox.v1.C1I1	cis.CUGBP.n2.I2	cis.CUGBP.n2.I2	cis.CUGBP.n3.I2
cisCons.Nova.NISE2	cis.CUGBP.n3.I2	cis.CUGBP.n3.I2	cis.MBNL.v2.I2
cisCons.Nova.NISS1	cis.Quak.I2	cis.MBNL.v3.I2	Len.C1
cisCons.Nova.NISS2	cis.MBNL.v2.I2	cis.PTB.v3.I2	cis.MBNL.v3.I2
cisCons.UGrich.v2.I2	cis.MBNL.v3.I2	Len.A	cis.Grich.I2

cisCons.CELFlike.I2	cis.Trich.WithC.I2	cis.Fox.v2.I1	cis.PTB.v1.I2
cisCons.Quak.I2	cis.PTB.v2.I2	cis.GUrich.v1.I1	cis.PTB.v2.I2
cisCons.MBNL.v3.I2	cis.GUrich.v1.I1	cis.Tra2.I1	Len.A
cisCons.Fox.v1.I2	cis.GUrich.v2.I1	cis.CUGBP.n2.I1	cis.Fox.v2.I1
cisCons.PTB.v2.I2	cis.Tra2.I1	cis.MBNL.v1.I1	cis.Fox.v3.I1
Frame.noShift.NaN	Len.C2	cis.MBNL.v3.I1	cis.GUrich.v1.I1
AltAGpos	cis.CUGBP.n2.I1	cis.PTB.v1.I1	cis.GUrich.v4.I1
cisCons.YeoClustDown.20.TGCATG.I2	cis.MBNL.v2.I1	cis.PTB.v2.I1	cis.CUGBP.n3.I1
cisCons.YeoClustDown.29.ACTAAC.I2	cis.PTB.v2.I1	cis.PTB.v3.I1	cis.PTB.v2.I1
Cons.MeanP1_100.I1	cis.PTB.v3.I1	cis.PTB.v4.I1	Len.I1
cisCons.YeoClustDown.47.GTACTGT.I2	cisCons.Nova.AI2_C2I2	cisCons.Nova.AI2_C2I2	cis.PTB.v4.I1
cisCons.YeoClustDown.50.GCATG.I2	cisCons.Nova.C1I1_AI1	cisCons.CUGBP.n3.I1I2	cisCons.Nova.C1I1_AI1
Cons.MeanP1_100.I2	cisCons.CUGBP.n2.I1I2	cisCons.PTB.v3.I1I2	cisCons.Nova.NISE3
cisCons.YeoClustDown.53.CTGACT.I2	cisCons.Nova.I1I2	cisCons.PTB.v4.I1I2	cisCons.CUGBP.n2.I1I2
cisCons.YeoClustDown.57.TTGGTT.I2	cisCons.Fox.v1.I1I2	cisCons.Nova.NISE2	cisCons.Nova.I1I2
cisCons.YeoClustDown.66.TTCACAG.I2	cisCons.PTB.v2.I1I2	cisCons.Nova.NISS1	cisCons.Fox.v1.I1I2
cisCons.YeoClustDown.68.TCTGG.I2	cisCons.PTB.v3.I1I2	cisCons.Nova.NISS2	cisCons.PTB.v2.I1I2
cisCons.YeoClustDown.73.GTGAG.I2	cisCons.Fox.v3.C1I1	cisCons.GUrich.v1.I2	cisCons.PTB.v3.I1I2
cisCons.YeoClustDown.74.TTCTGT.I2	cisCons.Nova.NISS1	Len.Ratio.A_I2	cisCons.Nova.NESE1_NESS2
SecStr.maxJunc.AI1	Len.Ratio.A_I1	cisCons.CUGBP.n3.I2	cisCons.Fox.v2.C1I1
cisCons.YeoClustDown.97.AAATT.I2	cisCons.Fox.v1.C2I2	cisCons.MBNL.v1.I2	cisCons.Nova.NISE2
cisCons.YeoClustDown.99.TTCTCT.I2	cisCons.Nova.NISS2	cisCons.MBNL.v2.I2	cisCons.Nova.NISE1
cisCons.YeoClustDown.103.TGTTTAA.I2	cisCons.Fox.v2.I2	cisCons.MBNL.v3.I2	cisCons.Nova.NISS1
SecStr.avgJunc.AI2	cisCons.GUrich.v1.I2	cisCons.Grich.I2	Len.Ratio.A_I1
cisCons.YeoClustDown.115.TGTCT.I2	cisCons.GUrich.v2.I2	cisCons.Trich.WithG.I2	cisCons.Fox.v1.C2I2
cisCons.YeoClustDown.122.GCTTCT.I2	cisCons.GUrich.v3.I2	cisCons.PTB.v2.I2	cisCons.Nova.NISS2
cisCons.YeoClustDown.123.TTCTAA.I2	cisCons.UGrich.v2.I2	Frame.noShift.NaN	cisCons.GUrich.v2.I2
cisCons.YeoClustDown.127.TGCTAA.I2	cisCons.CUGBP.n2.I2	AltAGpos	cisCons.Tra2.I2
cisCons.YeoClustDown.141.AGTAT.I2	cisCons.CUGBP.n3.I2	cisCons.YeoClustDown.1.GTAAC.I2	cisCons.CUGBP.n2.I2
cisCons.YeoClustDown.143.TGCCTTT.I2	cisCons.CELFlike.I2	cisCons.YeoClustDown.8.AATTG.I2	cisCons.CUGBP.n3.I2
cisCons.YeoClustDown.151.CTTTCA.I2	cisCons.Quak.I2	cisCons.YeoClustDown.9.CTGCT.I2	cisCons.Quak.I2
cisCons.YeoClustDown.157.TCTGC.I2	cisCons.MBNL.v1.I2	cisCons.YeoClustDown.19.TCATTTT.I2	cisCons.MBNL.v1.I2
cisCons.CUGBP.n2.I1	cisCons.MBNL.v2.I2	cisCons.YeoClustDown.20.TGCATG.I2	cisCons.MBNL.v2.I2
cisCons.MBNL.v3.I1	cisCons.MBNL.v3.I2	cisCons.YeoClustDown.25.ATAATT.I2	cisCons.MBNL.v3.I2
cisCons.Sugnet.I1	Len.Ratio.I1_I2	cisCons.YeoClustDown.27.GTATCCT.I2	Len.Ratio.I1_I2
cisCons.PTB.v2.I1	cisCons.Nova.I2	Cons.MeanP1_100.C2I2	cisCons.Nova.I2
cisCons.YeoClustUp.5.TAACC.I1	cisCons.Trich.WithC.I2	cisCons.YeoClustDown.32.TTAGCA.I2	cisCons.Grich.I2
cisCons.YeoClustUp.22.CTAAC.I1	cisCons.Fox.v1.I2	cisCons.YeoClustDown.34.TAATG.I2	cisCons.Fox.v1.I2
cisCons.YeoClustUp.24.TCTTC.I1	cisCons.PTB.v2.I2	cisCons.YeoClustDown.35.TTTTGAT.I2	cisCons.PTB.v1.I2
cisCons.YeoClustUp.25.TGCATG.I1	cisCons.PTB.v3.I2	cisCons.YeoClustDown.38.TATTTC.I2	cisCons.PTB.v3.I2
cisCons.YeoClustUp.28.TTTATC.I1	cisCons.PTB.v4.I2	cisCons.YeoClustDown.39.TAACT.I2	Frame.noShift.NaN
cisCons.YeoClustUp.34.TTTCAG.I1	Frame.noShift.NaN	cisCons.YeoClustDown.40.TGAGG.I2	AltAGpos
cisCons.YeoClustUp.45.TTCAAAA.I1	AltAGpos	cisCons.YeoClustDown.43.TATCCT.I2	cisCons.YeoClustDown.2.AAGTGT.I2
cisCons.YeoClustUp.55.GCTTTGC.I1	cisCons.YeoClustDown.2.AAGTGT.I2	cisCons.YeoClustDown.47.GTACTGT.I2	cisCons.YeoClustDown.4.ATTAACA.I2
cisCons.YeoClustUp.58.ACTAAT.I1	cisCons.YeoClustDown.3.GTTTGT.I2	cisCons.YeoClustDown.48.TTAAG.I2	cisCons.YeoClustDown.7.TTGAAAT.I2
cisCons.YeoClustUp.64.TCTTT.I1	cisCons.YeoClustDown.6.TAACC.I2	cisCons.YeoClustDown.49.CATAAA.I2	cisCons.YeoClustDown.8.AATTG.I2
cisCons.YeoClustUp.68.CTCTG.I1	cisCons.YeoClustDown.7.TTGAAAT.I2	cisCons.YeoClustDown.50.GCATG.I2	cisCons.YeoClustDown.9.CTGCT.I2
cisCons.YeoClustUp.71.TTTTCC.I1	cisCons.YeoClustDown.10.TTTATG.I2	Cons.MeanP1_100.I2	AltGTpos

cisCons.YeoClustUp.72.TTTTGC.I1	AltGTpos	cisCons.YeoClustDown.54.ACTAAT.I2	cisCons.YeoClustDown.12.ATGTTT.I2
cisCons.YeoClustUp.80.TGGCTT.I1	cisCons.YeoClustDown.13.TTTCCAA.I2	cisCons.YeoClustDown.55.GAGTA.I2	cisCons.YeoClustDown.16.GTACGT.I2
cisCons.YeoClustUp.91.ATTAAAT.I1	cisCons.YeoClustDown.14.AAGTC.I2	cisCons.YeoClustDown.56.TCTTAA.I2	cisCons.YeoClustDown.17.GTTAAA.I2
SecStr.avg1_70.AI1	cisCons.YeoClustDown.16.GTACGT.I2	cisCons.YeoClustDown.59.AGAGCCA.I2	cisCons.YeoClustDown.20.TGCATG.I2
cisCons.YeoClustUp.99.TTTAC.I1	cisCons.YeoClustDown.19.TCATTTT.I2	SecStr.maxJunc.C1I1	cisCons.YeoClustDown.22.TTTATC.I2
SecStr.max71_140.AI1	cisCons.YeoClustDown.20.TGCATG.I2	cisCons.YeoClustDown.62.GTATTT.I2	cisCons.YeoClustDown.25.ATAATT.I2
cisCons.YeoClustUp.102.TTCTAG.I1	Cons.MeanP1_100.C1I1	cisCons.YeoClustDown.68.TCTGG.I2	cisCons.YeoClustDown.29.ACTAAC.I2
cisCons.YeoClustUp.118.TCTCT.I1	cisCons.YeoClustDown.21.TTCTT.I2	cisCons.YeoClustDown.70.TGATTTG.I2	cisCons.YeoClustDown.31.AATTGA.I2
cisCons.YeoClustUp.121.TGTCT.I1	cisCons.YeoClustDown.23.ACATTT.I2	cisCons.YeoClustDown.73.GTGAG.I2	cisCons.YeoClustDown.32.TTAGCA.I2
cisCons.YeoClustUp.122.TTCCTT.I1	cisCons.YeoClustDown.24.TGCCAGC.I2	cisCons.YeoClustDown.74.TTCTGT.I2	cisCons.YeoClustDown.33.CAAAT.I2
cisCons.YeoClustUp.132.TCTGTT.I1	cisCons.YeoClustDown.26.GTAGG.I2	cisCons.YeoClustDown.79.TGGCTT.I2	cisCons.YeoClustDown.34.TAATG.I2
cisCons.YeoClustUp.148.TTGCTG.I1	cisCons.YeoClustDown.29.ACTAAC.I2	cisCons.YeoClustDown.80.TTAATCT.I2	cisCons.YeoClustDown.39.TAACT.I2
cisCons.YeoClustUp.152.TGCTT.I1	cisCons.YeoClustDown.32.TTAGCA.I2	SecStr.maxJunc.AI1	cisCons.YeoClustDown.40.TGAGG.I2
cis.ShortSeq.AG.C1	cisCons.YeoClustDown.33.CAAAT.I2	cisCons.YeoClustDown.81.AATTAT.I2	Cons.MeanP1_100.I1
cis.ShortSeq.CA.C1	cisCons.YeoClustDown.35.TTTTGAT.I2	cisCons.YeoClustDown.85.GCAAGT.I2	cisCons.YeoClustDown.42.TTTATA.I2
cis.ESS.Burge.C1	cisCons.YeoClustDown.39.TAACT.I2	cisCons.YeoClustDown.87.GTCTG.I2	cisCons.YeoClustDown.43.TATCCT.I2
cis.ShortSeq.CAG.C1	Cons.MeanP1_100.I1	cisCons.YeoClustDown.89.TGCAT.I2	cisCons.YeoClustDown.45.TTTACAG.I2
PTC.UponInclusion.NaN	cisCons.YeoClustDown.44.GTTAGT.I2	SecStr.avgJunc.AI1	cisCons.YeoClustDown.48.TTAAG.I2
cis.ESS.Burge.C2	cisCons.YeoClustDown.45.TTTACAG.I2	cisCons.YeoClustDown.93.GTTTT.I2	cisCons.YeoClustDown.49.CATAAA.I2
cis.ShortSeq.CTT.C1	cisCons.YeoClustDown.46.TATTTG.I2	cisCons.YeoClustDown.94.GCTTGGC.I2	cisCons.YeoClustDown.50.GCATG.I2
cis.ShortSeq.GTA.C1	cisCons.YeoClustDown.48.TTAAG.I2	cisCons.YeoClustDown.99.TTCTCT.I2	Cons.MeanP1_100.I2
cis.ShortSeq.TTT.C1	cisCons.YeoClustDown.50.GCATG.I2	cisCons.YeoClustDown.100.TGAGAA.I2	cisCons.YeoClustDown.57.TTGGTT.I2
cis.ShortSeq.TA.C2	Cons.MeanP1_100.I2	PTC.NotMined.NaN	cisCons.YeoClustDown.59.AGAGCCA.I2
cis.ShortSeq.CAT.C2	cisCons.YeoClustDown.51.TGATTA.I2	SecStr.maxJunc.AI2	SecStr.maxJunc.C1I1
cis.ShortSeq.CCA.C2	cisCons.YeoClustDown.53.CTGACT.I2	cisCons.YeoClustDown.103.TGTTTAA.I2	cisCons.YeoClustDown.63.TCAGA.I2
PTC.UponeExclusion.NaN	cisCons.YeoClustDown.54.ACTAAT.I2	cisCons.YeoClustDown.104.GTCAGT.I2	cisCons.YeoClustDown.67.AGTAA.I2
cis.ShortSeq.TCG.C2	cisCons.YeoClustDown.57.TTGGTT.I2	SecStr.avgJunc.AI2	cisCons.YeoClustDown.68.TCTGG.I2
cis.ShortSeq.CA.A	cisCons.YeoClustDown.60.TCTTT.I2	cisCons.YeoClustDown.111.TAATTTG.I2	cisCons.YeoClustDown.70.TGATTTG.I2
cis.ShortSeq.CC.A	SecStr.maxJunc.C1I1	cisCons.YeoClustDown.112.GAAATA.I2	SecStr.avgJunc.C1I1
cis.ShortSeq.CG.A	cisCons.YeoClustDown.62.GTATTT.I2	cisCons.YeoClustDown.115.TGTCT.I2	cisCons.YeoClustDown.71.TTTTGC.I2
cis.ShortSeq.GA.A	cisCons.YeoClustDown.66.TTCACAG.I2	cisCons.YeoClustDown.119.TATGT.I2	cisCons.YeoClustDown.72.TAGAAA.I2
cis.ShortSeq.TG.A	cisCons.YeoClustDown.68.TCTGG.I2	cisCons.YeoClustDown.122.GCTTCT.I2	cisCons.YeoClustDown.73.GTGAG.I2
cis.ShortSeq.AAT.A	cisCons.YeoClustDown.74.TTCTGT.I2	cisCons.YeoClustDown.125.AGATTT.I2	cisCons.YeoClustDown.75.GTAAG.I2
cis.ShortSeq.ACA.A	cisCons.YeoClustDown.79.TGGCTT.I2	cisCons.YeoClustDown.126.GAAAAT.I2	cisCons.YeoClustDown.78.TGAGC.I2
cis.ShortSeq.ACT.A	cisCons.YeoClustDown.80.TTAATCT.I2	cisCons.YeoClustDown.129.CTTTGCT.I2	cisCons.YeoClustDown.79.TGGCTT.I2
cis.ShortSeq.AGA.A	cisCons.YeoClustDown.83.CCACAG.I2	cisCons.YeoClustDown.131.TTTCTC.I2	cisCons.YeoClustDown.80.TTAATCT.I2
cis.ShortSeq.ATG.A	cisCons.YeoClustDown.85.GCAAGT.I2	cisCons.YeoClustDown.133.TTTGCC.I2	SecStr.maxJunc.AI1
cis.ShortSeq.CAT.A	cisCons.YeoClustDown.87.GTCTG.I2	cisCons.YeoClustDown.135.TGTTCT.I2	cisCons.YeoClustDown.82.TGGAAAT.I2
cis.ShortSeq.CCA.A	cisCons.YeoClustDown.89.TGCAT.I2	cisCons.YeoClustDown.139.TTGCAG.I2	cisCons.YeoClustDown.83.CCACAG.I2
cis.ShortSeq.CCC.A	cisCons.YeoClustDown.90.GAGAAA.I2	cisCons.YeoClustDown.143.TGCCTTT.I2	cisCons.YeoClustDown.84.AAATGA.I2
cis.ShortSeq.GCA.A	cisCons.YeoClustDown.94.GCTTGGC.I2	cisCons.YeoClustDown.145.GAGTG.I2	cisCons.YeoClustDown.85.GCAAGT.I2
cis.ShortSeq.GCT.A	cisCons.YeoClustDown.97.AAATT.I2	SecStr.avg1_70.C1I1	cisCons.YeoClustDown.86.GTAAAA.I2
cis.ShortSeq.TCT.A	cisCons.YeoClustDown.99.TTCTCT.I2	cisCons.YeoClustDown.151.CTTTCA.I2	cisCons.YeoClustDown.87.GTCTG.I2
JuncScore.AI1	cisCons.YeoClustDown.100.TGAGAA.I2	cisCons.YeoClustDown.152.AAGAT.I2	cisCons.YeoClustDown.89.TGCAT.I2
cis.ShortSeq.TGT.A	SecStr.maxJunc.AI2	cisCons.YeoClustDown.154.GTGGGT.I2	SecStr.avgJunc.AI1
cis.ShortSeq.TTC.A	cisCons.YeoClustDown.101.TTAGTT.I2	cisCons.YeoClustDown.155.GTAAAG.I2	cisCons.YeoClustDown.94.GCTTGGC.I2
cis.ShortSeq.TTG.A	cisCons.YeoClustDown.103.TGTTTAA.I2	cisCons.YeoClustDown.158.CTAAA.I2	cisCons.YeoClustDown.96.GTATG.I2
cis.ShortSeq.C.I2	cisCons.YeoClustDown.104.GTCAGT.I2	SecStr.max71_140.C1I1	cisCons.YeoClustDown.98.GTAAT.I2

cis.ShortSeq.AG.I2	cisCons.YeoClustDown.108.TCCTTT.I2	cisCons.GUrich.v3.I1	cisCons.YeoClustDown.100.TGAGAA.I2
cis.ShortSeq.CT.I2	SecStr.avgJunc.AI2	cisCons.UGrich.v2.I1	PTC.NotMined.NaN
cis.ShortSeq.ACT.I2	cisCons.YeoClustDown.115.TGTCT.I2	cisCons.MBNL.v1.I1	cisCons.YeoClustDown.101.TTAGTT.I2
cis.ShortSeq.GCT.I2	cisCons.YeoClustDown.116.GTTGGT.I2	SecStr.avg71_140.C1I1	cisCons.YeoClustDown.104.GTCAGT.I2
cis.PTB.v2.I1I2	cisCons.YeoClustDown.123.TTCTAA.I2	cisCons.MBNL.v2.I1	cisCons.YeoClustDown.106.TAAATG.I2
cis.ShortSeq.TGC.I2	cisCons.YeoClustDown.124.GTTTCT.I2	cisCons.Nova.I1	SecStr.avgJunc.AI2
cis.PTB.v4.I1I2	cisCons.YeoClustDown.128.AAGCT.I2	cisCons.Sugnet.I1	cisCons.YeoClustDown.111.TAATTTG.I2
JuncScore.AI2	cisCons.YeoClustDown.129.CTTTGCT.I2	cisCons.PTB.v1.I1	cisCons.YeoClustDown.112.GAAATA.I2
cis.ShortSeq.GA.I1	cisCons.YeoClustDown.130.TCTGA.I2	cisCons.PTB.v3.I1	cisCons.YeoClustDown.113.TGGTTT.I2
cis.ShortSeq.TCC.I1	SecStr.avgJunc.C2I2	cisCons.PTB.v4.I1	cisCons.YeoClustDown.114.TGTTAA.I2
cis.ShortSeq.TGC.I1	cisCons.YeoClustDown.131.TTTCTC.I2	cisCons.YeoClustUp.2.TCTCC.I1	cisCons.YeoClustDown.115.TGTCT.I2
cis.Nova.NISE2	cisCons.YeoClustDown.133.TTTGCC.I2	cisCons.YeoClustUp.5.TAACC.I1	cisCons.YeoClustDown.117.TGAATT.I2
cis.ShortSeq.CG.C1I1	cisCons.YeoClustDown.136.CTTTT.I2	cisCons.YeoClustUp.7.AAGCCA.I1	cisCons.YeoClustDown.118.AATTTA.I2
cis.ShortSeq.GA.C2I2	cisCons.YeoClustDown.139.TTGCAG.I2	cisCons.YeoClustUp.8.AATTG.I1	cisCons.YeoClustDown.119.TATGT.I2
	SecStr.max1_70.C1I1	cisCons.YeoClustUp.14.TCCAG.I1	cisCons.YeoClustDown.120.GCATTT.I2
	cisCons.YeoClustDown.143.TGCCTTT.I2	cisCons.YeoClustUp.20.CTTGAC.I1	cisCons.YeoClustDown.122.GCTTCT.I2
	cisCons.YeoClustDown.149.TGATTTT.I2	cisCons.YeoClustUp.21.TTAAAAC.I1	cisCons.YeoClustDown.125.AGATTT.I2
	SecStr.avg1_70.C1I1	SecStr.max1_70.AI2	cisCons.YeoClustDown.127.TGCTAA.I2
	cisCons.YeoClustDown.151.CTTTCA.I2	cisCons.YeoClustUp.22.CTAAC.I1	cisCons.YeoClustDown.128.AAGCT.I2
	cisCons.YeoClustDown.152.AAGAT.I2	cisCons.YeoClustUp.23.AAAGCT.I1	cisCons.YeoClustDown.129.CTTTGCT.I2
	cisCons.YeoClustDown.153.TGCTT.I2	cisCons.YeoClustUp.24.TCTTC.I1	SecStr.avgJunc.C2I2
	cisCons.YeoClustDown.154.GTGGGT.I2	cisCons.YeoClustUp.26.TTCTT.I1	cisCons.YeoClustDown.133.TTTGCC.I2
	cisCons.YeoClustDown.155.GTAAAG.I2	cisCons.YeoClustUp.28.TTTATC.I1	cisCons.YeoClustDown.135.TGTTCT.I2
	cisCons.YeoClustDown.157.TCTGC.I2	cisCons.YeoClustUp.38.TTTAAC.I1	cisCons.YeoClustDown.139.TTGCAG.I2
	cisCons.Fox.v2.I1	cisCons.YeoClustUp.40.AGAAAT.I1	cisCons.YeoClustDown.140.TAATA.I2
	cisCons.GUrich.v3.I1	cisCons.YeoClustUp.43.TAAAAT.I1	SecStr.max1_70.C1I1
	cisCons.MBNL.v1.I1	cisCons.YeoClustUp.47.CTTGTC.I1	cisCons.YeoClustDown.141.AGTAT.I2
	cisCons.MBNL.v2.I1	cisCons.YeoClustUp.49.TGGATTT.I1	cisCons.YeoClustDown.142.ATTCT.I2
	cisCons.MBNL.v3.I1	cisCons.YeoClustUp.51.TTAAG.I1	cisCons.YeoClustDown.143.TGCCTTT.I2
	cisCons.Sugnet.I1	cisCons.YeoClustUp.52.TTGGT.I1	cisCons.YeoClustDown.147.CTTTA.I2
	cisCons.PTB.v1.I1	cisCons.YeoClustUp.54.TGATTA.I1	cisCons.YeoClustDown.149.TGATTTT.I2
	cisCons.PTB.v2.I1	cisCons.YeoClustUp.58.ACTAAT.I1	SecStr.avg1_70.C1I1
	cisCons.PTB.v3.I1	cisCons.YeoClustUp.59.CTGACT.I1	cisCons.YeoClustDown.151.CTTTCA.I2
	SecStr.max141_210.C1I1	cisCons.YeoClustUp.63.TTTTGGC.I1	cisCons.YeoClustDown.154.GTGGGT.I2
	cisCons.YeoClustUp.2.TCTCC.I1	cisCons.YeoClustUp.64.TCTTT.I1	cisCons.YeoClustDown.157.TCTGC.I2
	cisCons.YeoClustUp.6.TTGAAAT.I1	cisCons.YeoClustUp.65.TTATTGA.I1	cisCons.YeoClustDown.158.CTAAA.I2
	cisCons.YeoClustUp.7.AAGCCA.I1	cisCons.YeoClustUp.66.TCAGA.I1	cisCons.Fox.v2.I1
	cisCons.YeoClustUp.9.CTGCT.I1	cisCons.YeoClustUp.69.TCTGG.I1	cisCons.Fox.v3.I1
	cisCons.YeoClustUp.10.TTTATG.I1	cisCons.YeoClustUp.70.AATTC.I1	cisCons.GUrich.v2.I1
	cisCons.YeoClustUp.11.TTCACA.I1	cisCons.YeoClustUp.72.TTTTGC.I1	cisCons.GUrich.v4.I1
	cisCons.YeoClustUp.12.TGATAA.I1	cisCons.YeoClustUp.74.TCCATTT.I1	cisCons.UGrich.v2.I1
	cisCons.YeoClustUp.15.TTTCCAA.I1	cisCons.YeoClustUp.76.CTTGATT.I1	cisCons.CUGBP.n2.I1
	cisCons.YeoClustUp.18.TCTTG.I1	cisCons.YeoClustUp.87.TCATTTC.I1	SecStr.avg71_140.C1I1
	cisCons.YeoClustUp.19.TTGTAA.I1	cisCons.YeoClustUp.92.CCTGCAG.I1	cisCons.MBNL.v2.I1
	cisCons.YeoClustUp.22.CTAAC.I1	cisCons.YeoClustUp.96.CTTCT.I1	cisCons.MBNL.v3.I1
	cisCons.YeoClustUp.23.AAAGCT.I1	cisCons.YeoClustUp.97.TTAGAA.I1	cisCons.Nova.I1
	cisCons.YeoClustUp.28.TTTATC.I1	cisCons.YeoClustUp.98.TGTTTC.I1	cisCons.Sugnet.I1
	cisCons.YeoClustUp.30.GCTGACC.I1	SecStr.max71_140.AI1	cisCons.Fox.v1.I1

	cisCons.YeoClustUp.35.TTAGCA.I1	cisCons.YeoClustUp.102.TTCTAG.I1	cisCons.PTB.v1.I1
	cisCons.YeoClustUp.36.TTGCCT.I1	cisCons.YeoClustUp.105.TGAGAA.I1	cisCons.PTB.v4.I1
	cisCons.YeoClustUp.38.TTTAAC.I1	cisCons.YeoClustUp.109.AAATCA.I1	cisCons.YeoClustUp.5.TAACC.I1
	cisCons.YeoClustUp.40.AGAAAT.I1	cisCons.YeoClustUp.111.CTTGC.I1	cisCons.YeoClustUp.7.AAGCCA.I1
	cisCons.YeoClustUp.41.TTTTGAT.I1	SecStr.avg71_140.AI1	cisCons.YeoClustUp.8.AATTG.I1
	cisCons.YeoClustUp.42.TAACT.I1	cisCons.YeoClustUp.118.TCTCT.I1	cisCons.YeoClustUp.9.CTGCT.I1
	cisCons.YeoClustUp.49.TGGATTT.I1	cisCons.YeoClustUp.121.TGTCT.I1	cisCons.YeoClustUp.12.TGATAA.I1
	cisCons.YeoClustUp.50.TTGCATT.I1	SecStr.max141_210.AI1	cisCons.YeoClustUp.14.TCCAG.I1
	cisCons.YeoClustUp.52.TTGGT.I1	cisCons.YeoClustUp.122.TTCCTT.I1	cisCons.YeoClustUp.15.TTTCCAA.I1
	cisCons.YeoClustUp.57.TTTTAAA.I1	cisCons.YeoClustUp.124.AATTTA.I1	cisCons.YeoClustUp.18.TCTTG.I1
	cisCons.YeoClustUp.61.CATTTA.I1	cisCons.YeoClustUp.125.GTTTCT.I1	PTC.ExcludedFromAnalysis.NaN
	cisCons.YeoClustUp.64.TCTTT.I1	cisCons.YeoClustUp.127.AGATTT.I1	cisCons.YeoClustUp.22.CTAAC.I1
	cisCons.YeoClustUp.66.TCAGA.I1	cisCons.YeoClustUp.128.GAAAAT.I1	cisCons.YeoClustUp.23.AAAGCT.I1
	cisCons.YeoClustUp.68.CTCTG.I1	cisCons.YeoClustUp.130.TTTGACT.I1	cisCons.YeoClustUp.24.TCTTC.I1
	cisCons.YeoClustUp.69.TCTGG.I1	cisCons.YeoClustUp.132.TCTGTT.I1	SecStr.avg1_70.AI2
	cisCons.YeoClustUp.72.TTTTGC.I1	cisCons.YeoClustUp.133.TTTATTC.I1	cisCons.YeoClustUp.33.CATTTG.I1
	cisCons.YeoClustUp.76.CTTGATT.I1	cisCons.YeoClustUp.134.TGAAAG.I1	cisCons.YeoClustUp.35.TTAGCA.I1
	cisCons.YeoClustUp.77.GTAAG.I1	cisCons.YeoClustUp.137.ATTTGT.I1	cisCons.YeoClustUp.36.TTGCCT.I1
	cisCons.YeoClustUp.79.AGAAAA.I1	cisCons.YeoClustUp.138.TGAGT.I1	cisCons.YeoClustUp.38.TTTAAC.I1
	cisCons.YeoClustUp.80.TGGCTT.I1	cisCons.YeoClustUp.139.CCCCAG.I1	cisCons.YeoClustUp.41.TTTTGAT.I1
	cisCons.YeoClustUp.82.TGGAAAT.I1	cisCons.YeoClustUp.140.TTGCAG.I1	cisCons.YeoClustUp.42.TAACT.I1
	cisCons.YeoClustUp.85.TCCTAG.I1	cisCons.YeoClustUp.142.TAATA.I1	cisCons.YeoClustUp.45.TTCAAAA.I1
	cisCons.YeoClustUp.89.AAATGA.I1	cisCons.YeoClustUp.143.TCTTA.I1	cisCons.YeoClustUp.48.TTTACAG.I1
	SecStr.avg1_70.AI1	cisCons.YeoClustUp.146.CTTTA.I1	cisCons.YeoClustUp.50.TTGCATT.I1
	cisCons.YeoClustUp.92.CCTGCAG.I1	cisCons.YeoClustUp.148.TTGCTG.I1	cisCons.YeoClustUp.51.TTAAG.I1
	cisCons.YeoClustUp.93.TTACAG.I1	cisCons.YeoClustUp.151.TTCTC.I1	SecStr.avg71_140.AI2
	cisCons.YeoClustUp.94.AAATGT.I1	cisCons.YeoClustUp.152.TGCTT.I1	cisCons.YeoClustUp.52.TTGGT.I1
	cisCons.YeoClustUp.95.TGCAT.I1	cisCons.YeoClustUp.155.GTAGGT.I1	cisCons.YeoClustUp.53.CATAAA.I1
	cisCons.YeoClustUp.96.CTTCT.I1	cis.PSSM.SF2_ASF.C1	cisCons.YeoClustUp.54.TGATTA.I1
	cisCons.YeoClustUp.99.TTTAC.I1	cis.PSSM.SRp55.C1	cisCons.YeoClustUp.55.GCTTTGC.I1
	cisCons.YeoClustUp.105.TGAGAA.I1	cis.PSSM.SF2_ASF.A	cisCons.YeoClustUp.56.ATTAG.I1
	cisCons.YeoClustUp.110.TGTTGA.I1	cis.PSSM.SRp40.A	cisCons.YeoClustUp.58.ACTAAT.I1
	cisCons.YeoClustUp.111.CTTGC.I1	cis.PSSM.SC35.C2	cisCons.YeoClustUp.60.ATATTT.I1
	cisCons.YeoClustUp.113.CCTCT.I1	cis.PSSM.SRp40.C2	cisCons.YeoClustUp.61.CATTTA.I1
	cisCons.YeoClustUp.116.TGTTAA.I1	cis.PSSM.SRp55.I2	SecStr.max141_210.AI2
	cisCons.YeoClustUp.118.TCTCT.I1	cis.ShortSeq.AT.C1	cisCons.YeoClustUp.62.AAATCT.I1
	cisCons.YeoClustUp.121.TGTCT.I1	cis.ShortSeq.CC.C1	cisCons.YeoClustUp.65.TTATTGA.I1
	cisCons.YeoClustUp.123.TGAATT.I1	cis.ShortSeq.GC.C1	cisCons.YeoClustUp.66.TCAGA.I1
	cisCons.YeoClustUp.125.GTTTCT.I1	cis.ShortSeq.TC.C1	cisCons.YeoClustUp.68.CTCTG.I1
	cisCons.YeoClustUp.129.GTTTAAT.I1	cis.ShortSeq.TG.C1	cisCons.YeoClustUp.69.TCTGG.I1
	SecStr.avg141_210.AI1	cis.ShortSeq.AGC.C1	cisCons.YeoClustUp.72.TTTTGC.I1
	cisCons.YeoClustUp.132.TCTGTT.I1	cis.ShortSeq.CAG.C1	cisCons.YeoClustUp.74.TCCATTT.I1
	cisCons.YeoClustUp.133.TTTATTC.I1	cis.ShortSeq.CCT.C1	cisCons.YeoClustUp.76.CTTGATT.I1
	cisCons.YeoClustUp.136.CTTTT.I1	PTC.UponInclusion.NaN	cisCons.YeoClustUp.77.GTAAG.I1
	cisCons.YeoClustUp.137.ATTTGT.I1	cis.ShortSeq.CTA.C1	cisCons.YeoClustUp.79.AGAAAA.I1
	cisCons.YeoClustUp.139.CCCCAG.I1	cis.ShortSeq.CTC.C1	cisCons.YeoClustUp.81.CTCAG.I1
	SecStr.max1_70.C2I2	cis.ShortSeq.CTT.C1	SecStr.max1_70.AI1
	cisCons.YeoClustUp.143.TCTTA.I1	cis.ShortSeq.GAA.C1	cisCons.YeoClustUp.82.TGGAAAT.I1

	cisCons.YeoClustUp.147.TTTAG.I1	cis.ShortSeq.GAC.C1	cisCons.YeoClustUp.83.AATTAT.I1
	cisCons.YeoClustUp.148.TTGCTG.I1	cis.ShortSeq.GAT.C1	cisCons.YeoClustUp.85.TCCTAG.I1
	cisCons.YeoClustUp.150.CTTTCA.I1	cis.ShortSeq.GCA.C1	cisCons.YeoClustUp.88.AAAGCA.I1
	cisCons.YeoClustUp.151.TTCTC.I1	cis.ShortSeq.GCC.C1	cisCons.YeoClustUp.92.CCTGCAG.I1
	cisCons.YeoClustUp.152.TGCTT.I1	cis.ShortSeq.GGA.C1	cisCons.YeoClustUp.93.TTACAG.I1
	cisCons.YeoClustUp.153.TCTGC.I1	cis.ESE.Burge.C1	cisCons.YeoClustUp.96.CTTCT.I1
	cisCons.YeoClustUp.155.GTAGGT.I1	cis.ShortSeq.TAC.C1	cisCons.YeoClustUp.101.CTTCCA.I1
	cis.PSSM.SC35.C1	cis.ShortSeq.TAG.C1	SecStr.max71_140.AI1
	cis.PSSM.SC35.C2	cis.ShortSeq.TAT.C1	cisCons.YeoClustUp.105.TGAGAA.I1
	cis.PSSM.SF2_ASF.C2	cis.ShortSeq.TGT.C1	cisCons.YeoClustUp.107.GTCAGT.I1
	cis.PSSM.SRp40.C2	cis.ShortSeq.TTC.C1	cisCons.YeoClustUp.111.CTTGC.I1
	cis.PSSM.SRp40.I1	cis.ShortSeq.TTG.C1	cisCons.YeoClustUp.113.CCTCT.I1
	cis.ShortSeq.C.C1	cis.ESS.Chasin.A	cisCons.YeoClustUp.116.TGTTAA.I1
	cis.ShortSeq.T.C1	cis.ShortSeq.T.C2	cisCons.YeoClustUp.118.TCTCT.I1
	cis.ShortSeq.CA.C1	cis.ShortSeq.AT.C2	cisCons.YeoClustUp.119.TTAACA.I1
	cis.ShortSeq.CC.C1	cis.ShortSeq.GC.C2	cisCons.YeoClustUp.120.TTTGGT.I1
	cis.ShortSeq.CT.C1	cis.ShortSeq.GT.C2	cisCons.YeoClustUp.123.TGAATT.I1
	cis.ShortSeq.GC.C1	cis.ShortSeq.TG.C2	cisCons.YeoClustUp.126.TGCTAA.I1
	SecStr.avg141_210.C2I2	cis.ShortSeq.ACA.C2	cisCons.YeoClustUp.128.GAAAAT.I1
	cis.ShortSeq.TC.C1	cis.ShortSeq.ACT.C2	cisCons.YeoClustUp.130.TTTGACT.I1
	cis.ShortSeq.TG.C1	cis.ShortSeq.AGC.C2	SecStr.avg141_210.AI1
	cis.ShortSeq.TT.C1	cis.ESE.Chasin.A	cisCons.YeoClustUp.132.TCTGTT.I1
	cis.ShortSeq.ACC.C1	cis.ShortSeq.ATC.C2	cisCons.YeoClustUp.138.TGAGT.I1
	cis.ShortSeq.AGC.C1	cis.ShortSeq.ATG.C2	cisCons.YeoClustUp.142.TAATA.I1
	cis.ShortSeq.ATA.C1	cis.ShortSeq.CAA.C2	cisCons.YeoClustUp.148.TTGCTG.I1
	cis.ShortSeq.ATC.C1	cis.ShortSeq.CCA.C2	cisCons.YeoClustUp.151.TTCTC.I1
	cis.ShortSeq.ATG.C1	cis.ShortSeq.CCC.C2	cisCons.YeoClustUp.155.GTAGGT.I1
	cis.ESS.Burge.C1	cis.ShortSeq.CTC.C2	cisCons.YeoClustUp.156.CTAAA.I1
	cis.ShortSeq.CAG.C1	cis.ShortSeq.CTG.C2	cis.PSSM.SC35.C1
	cis.ShortSeq.CAT.C1	cis.ShortSeq.GCA.C2	cis.PSSM.SRp55.C1
	cis.ShortSeq.CCC.C1	cis.ShortSeq.GGA.C2	cis.PSSM.SF2_ASF.A
	PTC.UponInclusion.NaN	PTC.UponeExclusion.NaN	cis.PSSM.SRp55.A
	cis.ESS.Burge.C2	cis.NovaClust.NESE1_NESS2	cis.PSSM.SF2_ASF.C2
	cis.ShortSeq.CGC.C1	cis.ShortSeq.GGG.C2	cis.PSSM.SRp40.C2
	cis.ShortSeq.CTA.C1	cis.ShortSeq.GTG.C2	cis.PSSM.SRp55.C2
	cis.ShortSeq.CTC.C1	cis.ShortSeq.GTT.C2	cis.PSSM.SRp40.I1
	cis.ESE.Burge.A	cis.ShortSeq.TAG.C2	cis.ShortSeq.C.C1
	cis.ShortSeq.GAT.C1	cis.ShortSeq.TTA.C2	cis.ShortSeq.AT.C1
	cis.ShortSeq.GCC.C1	cis.ShortSeq.TTG.C2	cis.ShortSeq.CA.C1
	cis.ShortSeq.GGC.C1	cis.ShortSeq.TTT.C2	cis.ShortSeq.GG.C1
	cis.ShortSeq.GGG.C1	cis.ShortSeq.AC.A	SecStr.avg141_210.C2I2
	cis.ShortSeq.GTA.C1	cis.ShortSeq.CT.A	cis.ShortSeq.GT.C1
	cis.ShortSeq.GTC.C1	cis.ShortSeq.GG.A	cis.ShortSeq.TA.C1
	cis.ShortSeq.GTT.C1	cis.ShortSeq.GT.A	cis.ShortSeq.TC.C1
	cis.ShortSeq.TAA.C1	cis.ShortSeq.TA.A	cis.ShortSeq.TT.C1
	cis.ShortSeq.TAG.C1	cis.ShortSeq.AAC.A	cis.ShortSeq.AAC.C1
	cis.ShortSeq.TAT.C1	cis.ShortSeq.ACC.A	cis.ShortSeq.AAG.C1
	cis.ShortSeq.TCG.C1	cis.ShortSeq.AGT.A	cis.ShortSeq.AAT.C1

	cis.ESE.Burge.C2	cis.ShortSeq.ATC.A	cis.ShortSeq.ACA.C1
	cis.ShortSeq.TGA.C1	cis.ShortSeq.CAC.A	cis.ESS.Burge.A
	cis.ShortSeq.TGC.C1	cis.ShortSeq.CAT.A	cis.ShortSeq.ACC.C1
	cis.ShortSeq.TGT.C1	cis.ShortSeq.CCC.A	cis.ShortSeq.ACT.C1
	cis.ShortSeq.TTG.C1	cis.ShortSeq.CCG.A	cis.ShortSeq.AGA.C1
	cis.ShortSeq.C.C2	cis.ShortSeq.CGA.A	cis.ShortSeq.AGC.C1
	cis.ShortSeq.CA.C2	cis.ShortSeq.CGC.A	cis.ShortSeq.ATC.C1
	cis.ShortSeq.CT.C2	cis.ShortSeq.CTA.A	cis.ShortSeq.ATG.C1
	cis.ShortSeq.GT.C2	cis.ShortSeq.CTC.A	cis.ESS.Burge.C1
	cis.ShortSeq.TA.C2	cis.ShortSeq.CTG.A	cis.ShortSeq.ATT.C1
	cis.ShortSeq.TC.C2	cis.ShortSeq.GAA.A	cis.ShortSeq.CAA.C1
	cis.ShortSeq.AAG.C2	cis.ShortSeq.GAG.A	cis.ShortSeq.CAG.C1
	cis.ShortSeq.AAT.C2	cis.ShortSeq.GAT.A	cis.ShortSeq.CCA.C1
	cis.ShortSeq.ACA.C2	cis.ShortSeq.GCA.A	cis.ShortSeq.CCT.C1
	cis.ShortSeq.ACC.C2	cis.ShortSeq.GCG.A	PTC.UponInclusion.NaN
	cis.ShortSeq.ACT.C2	cis.ShortSeq.GCT.A	cis.ShortSeq.GAA.C1
	cis.ShortSeq.AGG.C2	cis.ShortSeq.GGG.A	cis.ShortSeq.GAG.C1
	cis.ShortSeq.CAG.C2	cis.ShortSeq.GTG.A	cis.ESE.Burge.A
	cis.ShortSeq.CAT.C2	cis.ShortSeq.GTT.A	cis.ShortSeq.GAT.C1
	cis.ShortSeq.CCC.C2	cis.ShortSeq.TAG.A	cis.ShortSeq.GCA.C1
	cis.ShortSeq.CGA.C2	cis.ShortSeq.TCC.A	cis.ShortSeq.GCT.C1
	cis.ShortSeq.CGC.C2	cis.ShortSeq.TCG.A	cis.ShortSeq.GGG.C1
	cis.ShortSeq.CGG.C2	cis.ShortSeq.TGA.A	cis.ShortSeq.GGT.C1
	cis.ShortSeq.CTA.C2	JuncScore.AI1	cis.ShortSeq.GTA.C1
	cis.ShortSeq.CTC.C2	cis.Nova.C1	cis.ESE.Burge.C1
	cis.ESE.Chasin.C2	cis.ShortSeq.TGT.A	cis.ShortSeq.GTT.C1
	cis.ShortSeq.GAC.C2	cis.ShortSeq.TTG.A	cis.ShortSeq.TAC.C1
	cis.ShortSeq.GCC.C2	cis.ShortSeq.TTT.A	cis.ShortSeq.TAG.C1
	cis.ShortSeq.GGA.C2	cis.ShortSeq.A.I2	cis.ShortSeq.TAT.C1
	PTC.UponeExclusion.NaN	cis.Nova.NISE3	cis.ShortSeq.TCA.C1
	cis.ShortSeq.GGT.C2	cis.ShortSeq.AC.I2	cis.ShortSeq.TCC.C1
	cis.ShortSeq.TAC.C2	cis.ShortSeq.CA.I2	cis.ShortSeq.TCT.C1
	cis.NovaClust.NESS1	cis.ShortSeq.CG.I2	cis.ShortSeq.TGA.C1
	cis.ShortSeq.TCG.C2	cis.ShortSeq.CT.I2	cis.ShortSeq.TGG.C1
	cis.ShortSeq.TGC.C2	cis.CUGBP.n2.I1I2	cis.ShortSeq.TTA.C1
	cis.ShortSeq.TGT.C2	cis.ShortSeq.TC.I2	cis.ShortSeq.A.C2
	cis.NovaClust.NISE1	cis.ShortSeq.TG.I2	cis.ShortSeq.AT.C2
	cis.ShortSeq.TTC.C2	cis.ShortSeq.AAA.I2	cis.ESS.Chasin.C1
	cis.ShortSeq.TTT.C2	cis.ShortSeq.AAC.I2	cis.ShortSeq.TA.C2
	cis.ShortSeq.AG.A	cis.CUGBP.n3.I1I2	cis.ShortSeq.TT.C2
	cis.NovaClust.NISE2	cis.ShortSeq.ACG.I2	cis.ShortSeq.AAT.C2
	cis.ShortSeq.CA.A	cis.ShortSeq.AGC.I2	cis.ShortSeq.ACC.C2
	cis.ShortSeq.CC.A	cis.ShortSeq.ATC.I2	cis.ShortSeq.ACG.C2
	cis.ShortSeq.CG.A	cis.ShortSeq.CAC.I2	cis.ShortSeq.AGA.C2
	cis.ShortSeq.CT.A	cis.ShortSeq.CGA.I2	cis.ShortSeq.AGG.C2
	cis.ShortSeq.GA.A	cis.ShortSeq.CGT.I2	cis.ShortSeq.ATC.C2
	cis.ShortSeq.TA.A	cis.PTB.v1.I1I2	cis.ShortSeq.ATT.C2
	cis.ShortSeq.AAA.A	cis.ShortSeq.GAC.I2	cis.ShortSeq.CAA.C2

	cis.ShortSeq.AAT.A	cis.ShortSeq.GCT.I2	cis.ShortSeq.CAG.C2
	cis.ShortSeq.ACA.A	cis.ShortSeq.GTT.I2	cis.ShortSeq.CAT.C2
	cis.ShortSeq.ACG.A	cis.ShortSeq.TAG.I2	cis.ShortSeq.CCA.C2
	cis.ShortSeq.ACT.A	cis.ShortSeq.TCA.I2	cis.ShortSeq.CCC.C2
	cis.ShortSeq.ATG.A	cis.ShortSeq.TCT.I2	cis.ShortSeq.CCG.C2
	cis.ShortSeq.ATT.A	cis.ShortSeq.TGA.I2	cis.ShortSeq.CGG.C2
	cis.ShortSeq.CCC.A	cis.ShortSeq.TGT.I2	cis.ShortSeq.CTA.C2
	cis.ShortSeq.CCG.A	cis.ShortSeq.TTA.I2	cis.ShortSeq.CTC.C2
	cis.ShortSeq.CGC.A	cis.PTB.v4.I1I2	cis.ESE.Chasin.C2
	cis.Nova.NESS1	cis.ShortSeq.AC.I1	cis.ShortSeq.CTT.C2
	cis.ShortSeq.CTG.A	cis.ShortSeq.CA.I1	cis.ShortSeq.GAC.C2
	cis.ShortSeq.CTT.A	JuncScore.AI2	cis.ShortSeq.GCC.C2
	cis.ShortSeq.GAC.A	cis.ShortSeq.TC.I1	cis.ShortSeq.GCG.C2
	cis.ShortSeq.GTC.A	cis.ShortSeq.AAC.I1	cis.ShortSeq.GGA.C2
	cis.ShortSeq.TAG.A	cis.ShortSeq.AAG.I1	PTC.UponeExclusion.NaN
	cis.ShortSeq.TCG.A	cis.ShortSeq.ACA.I1	cis.NovaClust.NESE1_NESS2
	JuncScore.AI1	cis.ShortSeq.ACT.I1	cis.ShortSeq.GGC.C2
	cis.ShortSeq.TTC.A	cis.ShortSeq.AGC.I1	cis.ShortSeq.GGG.C2
	cis.ShortSeq.TTG.A	cis.ShortSeq.AGT.I1	cis.ShortSeq.GGT.C2
	cis.ShortSeq.A.I2	cis.ShortSeq.ATC.I1	cis.ShortSeq.GTT.C2
	cis.ShortSeq.GA.I2	cis.ShortSeq.CAA.I1	cis.ShortSeq.TAC.C2
	cis.ShortSeq.GC.I2	cis.ShortSeq.CAG.I1	cis.ShortSeq.TAG.C2
	cis.ShortSeq.GT.I2	cis.ShortSeq.CCC.I1	cis.NovaClust.NESS1
	cis.ShortSeq.ACA.I2	cis.ShortSeq.CGC.I1	cis.ShortSeq.TAT.C2
	cis.ShortSeq.ACG.I2	cis.ShortSeq.CTA.I1	cis.ShortSeq.TCA.C2
	cis.ShortSeq.AGA.I2	cis.Fox.v1.C1I1	cis.ShortSeq.TCC.C2
	cis.ShortSeq.AGG.I2	cis.Tra2.A	cis.ShortSeq.TCG.C2
	cis.ShortSeq.ATC.I2	cis.ShortSeq.TAC.I1	cis.ShortSeq.TGG.C2
	cis.ShortSeq.CAA.I2	cis.ShortSeq.TCT.I1	cis.ShortSeq.TTA.C2
	cis.ShortSeq.CAC.I2	cis.ShortSeq.TGG.I1	cis.ShortSeq.TTG.C2
	cis.ShortSeq.CAG.I2	cis.ShortSeq.TTG.I1	cis.ShortSeq.TTT.C2
	cis.ShortSeq.CCG.I2	cis.ShortSeq.AC.C1I1	cis.ShortSeq.A.A
	cis.ShortSeq.CGC.I2	cis.ShortSeq.CA.C1I1	cis.ShortSeq.T.A
	cis.ShortSeq.CGT.I2	cis.ShortSeq.CG.C1I1	cis.ShortSeq.AC.A
	cis.ShortSeq.CTT.I2	cis.Nova.NISE1	cis.ShortSeq.AG.A
	cis.ShortSeq.GAA.I2	cis.ShortSeq.GT.C1I1	cis.NovaClust.NISE2
	cis.ShortSeq.GAT.I2	cis.ShortSeq.TG.C1I1	cis.ShortSeq.AT.A
	cis.ShortSeq.GCG.I2	cis.ShortSeq.AA.C2I2	cis.ShortSeq.CA.A
	cis.ShortSeq.GGC.I2	cis.ShortSeq.AC.C2I2	cis.ShortSeq.CC.A
	cis.ShortSeq.GGT.I2	cis.ShortSeq.AG.C2I2	cis.ShortSeq.CG.A
	cis.ShortSeq.GTA.I2	cis.ShortSeq.GA.C2I2	cis.ShortSeq.CT.A
	cis.ShortSeq.GTC.I2	cis.ShortSeq.TC.C2I2	cis.ShortSeq.TA.A
	cis.ShortSeq.GTG.I2	cis.ShortSeq.TG.C2I2	cis.NovaClust.NISE3
	cis.ShortSeq.TAC.I2		cis.ShortSeq.TC.A
	cis.ShortSeq.TAG.I2		cis.ShortSeq.TG.A
	cis.ShortSeq.TCG.I2		cis.ShortSeq.TT.A
	cis.PTB.v4.I1I2		cis.ShortSeq.AAC.A
	cis.ShortSeq.A.I1		cis.ShortSeq.ACA.A

	cis.ShortSeq.C.I1	cis.ShortSeq.ACC.A
	cis.ShortSeq.AG.I1	cis.ShortSeq.ACT.A
	JuncScore.AI2	cis.ShortSeq.AGA.A
	cis.ShortSeq.GT.I1	cis.ShortSeq.AGC.A
	cis.ShortSeq.TC.I1	cis.ShortSeq.AGG.A
	cis.ShortSeq.AAC.I1	cis.ShortSeq.ATA.A
	cis.ShortSeq.AAG.I1	cis.ShortSeq.ATC.A
	cis.ShortSeq.ACA.I1	cis.ShortSeq.ATG.A
	cis.ShortSeq.AGA.I1	cis.ShortSeq.CAT.A
	cis.ShortSeq.AGT.I1	cis.ShortSeq.CCA.A
	cis.ShortSeq.CAA.I1	cis.ShortSeq.CCC.A
	cis.Fox.v3.C1I1	cis.ShortSeq.CGA.A
	cis.ShortSeq.CCC.I1	cis.ShortSeq.CTT.A
	cis.ShortSeq.CCG.I1	cis.ShortSeq.GAA.A
	cis.ShortSeq.CTA.I1	cis.ShortSeq.GAT.A
	cis.Fox.v1.C1I1	cis.ShortSeq.GCC.A
	cis.ShortSeq.CTC.I1	cis.ShortSeq.GCT.A
	cis.ShortSeq.GAA.I1	cis.ShortSeq.GGT.A
	cis.ShortSeq.GAC.I1	cis.ShortSeq.GTA.A
	cis.ShortSeq.GCG.I1	cis.ShortSeq.GTC.A
	cis.Tra2.A	cis.ShortSeq.GTG.A
	cis.ShortSeq.GTT.I1	cis.ShortSeq.TAC.A
	cis.Nova.A	cis.ShortSeq.TAG.A
	cis.ShortSeq.TAG.I1	cis.ShortSeq.TCA.A
	cis.ShortSeq.TCC.I1	cis.ShortSeq.TCC.A
	cis.ShortSeq.TCG.I1	cis.ShortSeq.TCT.A
	cis.ShortSeq.TCT.I1	JuncScore.AI1
	cis.ShortSeq.TGA.I1	cis.ShortSeq.TGT.A
	cis.ShortSeq.TGC.I1	cis.ShortSeq.TTG.A
	cis.ShortSeq.TGG.I1	cis.ShortSeq.T.I2
	cis.ShortSeq.TTC.I1	cis.ShortSeq.AC.I2
	cis.Nova.NISE2	cis.ShortSeq.CA.I2
	cis.ShortSeq.AG.C1I1	cis.ShortSeq.CG.I2
	cis.ShortSeq.CC.C1I1	cis.ShortSeq.CT.I2
	cis.ShortSeq.CT.C1I1	cis.ShortSeq.TC.I2
	cis.Nova.NISE1	cis.CUGBP.n3.I1I2
	cis.ShortSeq.GT.C1I1	cis.ShortSeq.ACA.I2
	cis.ShortSeq.TG.C1I1	cis.ShortSeq.ACC.I2
	cis.ShortSeq.TT.C1I1	cis.ShortSeq.ACT.I2
	cis.ShortSeq.CA.C2I2	cis.ShortSeq.AGA.I2
	cis.ShortSeq.CG.C2I2	cis.ShortSeq.AGC.I2
	cis.ShortSeq.GA.C2I2	cis.ShortSeq.AGG.I2
	cis.ShortSeq.TC.C2I2	cis.ShortSeq.AGT.I2
		cis.ShortSeq.ATA.I2
		cis.ShortSeq.ATG.I2
		cis.ShortSeq.CAA.I2
		cis.ShortSeq.CCC.I2
		cis.Fox.v1.I1I2

cis.ShortSeq.CGA.I2
cis.ShortSeq.CGC.I2
cis.ShortSeq.CGG.I2
cis.ShortSeq.CGT.I2
cis.ShortSeq.CTA.I2
cis.ShortSeq.GAA.I2
cis.ShortSeq.GAC.I2
cis.ShortSeq.GAT.I2
cis.ShortSeq.GCC.I2
cis.ShortSeq.GCT.I2
cis.ShortSeq.GGC.I2
cis.ShortSeq.GGT.I2
cis.ShortSeq.GTG.I2
cis.ShortSeq.GTT.I2
cis.ShortSeq.TAC.I2
cis.ShortSeq.TAG.I2
cis.ShortSeq.TCA.I2
cis.PTB.v3.I1I2
cis.ShortSeq.TCG.I2
cis.ShortSeq.TCT.I2
cis.ShortSeq.TGA.I2
cis.ShortSeq.TGC.I2
cis.ShortSeq.TGG.I2
cis.ShortSeq.TTA.I2
cis.ShortSeq.T.I1
cis.ShortSeq.AA.I1
cis.ShortSeq.AC.I1
JuncScore.AI2
cis.ShortSeq.CG.I1
cis.ShortSeq.GA.I1
cis.ShortSeq.AAC.I1
cis.ShortSeq.AAG.I1
cis.ShortSeq.ACC.I1
cis.ShortSeq.ACT.I1
cis.ShortSeq.AGA.I1
cis.Fox.v2.C1I1
cis.ShortSeq.AGC.I1
cis.ShortSeq.AGT.I1
cis.ShortSeq.ATC.I1
cis.ShortSeq.ATG.I1
cis.ShortSeq.CAC.I1
cis.Fox.v3.C1I1
cis.ShortSeq.CCA.I1
cis.ShortSeq.CCG.I1
cis.ShortSeq.CGC.I1
cis.ShortSeq.CGG.I1
cis.ShortSeq.CTA.I1
cis.ShortSeq.GAA.I1

cis.ShortSeq.GCG.I1
cis.Tra2.A
cis.ShortSeq.GCT.I1
cis.ShortSeq.GTC.I1
cis.ShortSeq.GTG.I1
cis.ShortSeq.TAC.I1
cis.ShortSeq.TAG.I1
cis.ShortSeq.TAT.I1
cis.ShortSeq.TGA.I1
cis.ShortSeq.TGC.I1
cis.ShortSeq.TTC.I1
cis.ShortSeq.AA.C1I1
cis.ShortSeq.CT.C1I1
cis.ShortSeq.GA.C1I1
cis.ShortSeq.TC.C1I1
cis.ShortSeq.AA.C2I2
cis.ShortSeq.AC.C2I2
cis.ShortSeq.CA.C2I2
cis.ShortSeq.CG.C2I2
cis.ShortSeq.CT.C2I2
